# Hemoglobin Digestion Genes Are Conserved in Lizard‐Infective *Plasmodium* Species With Different Host Cellular Niches

**DOI:** 10.1002/ece3.73801

**Published:** 2026-06-08

**Authors:** Sarah J. Pangburn, Janus Borner, Sidhanth Misra, Jessica Zavalunova, Susan L. Perkins

**Affiliations:** ^1^ Biology Program The Graduate Center at the City University of New York New York New York USA; ^2^ Department of Biology The City College of New York at the City University of New York New York New York USA; ^3^ Richard Gilder Graduate School American Museum of Natural History New York New York USA; ^4^ Institute for Comparative Genomics American Museum of Natural History New York New York USA

**Keywords:** comparative transcriptomics, hemoglobin digestion, malaria, *Plasmodium*

## Abstract

In their vertebrate hosts, malaria parasites typically inhabit erythrocytes where they harvest the cell's abundant supply of hemoglobin as a nutrient source. A byproduct of hemoglobin digestion is free heme, which the parasites detoxify by converting it to a brown crystal known as hemozoin. Hemozoin is a hallmark of *Plasmodium* infection, and this enzymatic pathway is well studied in mammalian *Plasmodium* species. Despite their evolutionary relatedness to mammalian *Plasmodium*, wildlife malaria parasites, particularly those that infect birds and lizards, are understudied, leaving their vast genetic diversity to be explored. *Plasmodium floridense*, *Plasmodium azurophilum*, and *Plasmodium leucocytica* infect *Anolis* lizards throughout the Caribbean islands, including the endemic anole on the island of Saba. Like other *Plasmodium* species, *P. floridense* infects red blood cells and produces hemozoin. *P. azurophilum* also infects red blood cells, however, its sister species, *P. leucocytica*, infects white blood cells. This is atypical for *Plasmodium* parasites and represents an expansion into a new cellular niche. Two of these three parasites (*P. azurophilum* and *P. leucocytica*) also do not produce hemozoin and have seemingly evolved alternative mechanisms of hemoglobin digestion for nutrient acquisition. To investigate this, we assembled parasite transcriptomes from infected Saban anole blood samples and analyzed them for hemoglobin digestion transcripts. The transcriptome results indicate that all three lizard parasites transcribe the genes canonically involved in the hemoglobin digestion pathway. This is the first evidence that these parasites possess genes for the same digestive enzymes as the better characterized mammalian *Plasmodium*, indicating conservation of this pathway across the *Plasmodium* tree. However, there is evidence for shifts in selective pressure on some of these proteins in all three lizard‐infective species. These genes may not be as functionally important relative to other *Plasmodium* species. Since the genes are not yet pseudogenes, however, there remains the alternative hypothesis that these genes also play additional roles in malaria parasite biology.

## Graphical abstract

Despite their evolutionary relatedness to malaria parasites that infect humans, wildlife *Plasmodium*—particularly those that infect birds and lizards—are understudied, leaving their vast genetic diversity to be explored. By assembling parasite transcriptomes from *Plasmodium*‐infected lizard blood, we provide the first evidence that lizard‐infective parasites possess and express conserved molecular pathways found in other malaria lineages. However, signatures of altered selective pressures on several of these genes suggest evolutionary shifts in their functional importance, highlighting how malaria parasite biology may change across vertebrate hosts, which we correlate to lineage‐specific niche partitioning within the infected host.

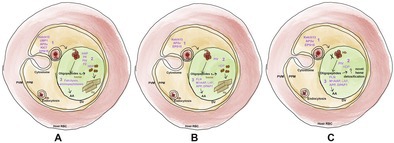

## 1 Introduction

**FIGURE 1:**
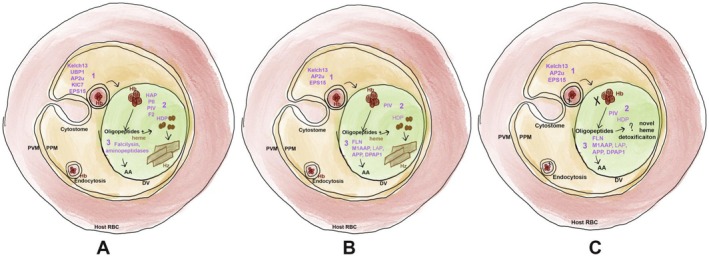
Hemoglobin digestion pathway in *Plasmodium* species. (A) Genes involved in the hemoglobin (Hb) uptake (1 on diagram) and digestion pathway (2, 3 on diagram) based on *Plasmodium falciparum* and *Plasmodium berghei* studies (Elliott et al. 2008; Birnbaum et al. 2020; Chugh et al. 2013). Hb uptake occurs via initial endocytosis (ring stage and digestive vacuole formation) and cytostome pathway (trophozoite stage and later). The labeled Kelch13 compartment on this diagram only includes the proteins found to impair endocytosis when inactivated (Birnbaum et al. 2020; Schmidt et al. 2023). Heme (byproduct from digestion) gets converted to heme dimers then hemozoin (Hz). Heme detoxification protein (HDP), histo aspartic protease (HAP), plasmepsin II (PII), plasmepsin IV (PIV), falcipain 2 (F2), parasitophorous vacuole membrane (PVM), parasite plasma membrane (PPM), digestive vacuole (DV), amino acids (AA). (B) Proposed hemoglobin digestion pathway for *Plasmodium floridense*. Only genes identified via transcriptomics are listed. Genes in bold font indicate shifts in selective pressure were identified for given gene. (C) Proposed protein digestion pathway for *Plasmodium azurophilum*. Only genes identified via transcriptomics are listed. Genes in bold font indicate shifts in selective pressure were identified for given gene. Possible digestion pathways include: Hemoglobin is imported into the host cell and a novel form of heme detoxification is occurring (not hemozoin formation), other host cell proteins labeled X on the diagram are digested (which would not require heme detoxification), or both scenarios are occurring. This proposed pathway may also apply to other non‐hemozoin forming red blood cell parasites such as *Babesia* species.

There were an estimated 249 million cases of malaria in 2022, causing over 600,000 deaths globally (WHO 2023). The World Health Organization currently recommends the use of artemisinin‐based combination therapies (ACTs) to treat malaria; however, the efficacy of ACTs is threatened by the emergence of multi‐drug resistance, now reported in multiple countries (WHO 2023). Malaria, therefore, is still a global health concern and new treatment options are needed. *Plasmodium* species, the causative agents of malaria, are often characterized by two hallmark features of their asexual stages: the invasion of vertebrate red blood cells and the presence of hemozoin pigment as a byproduct of host hemoglobin (Hb) digestion. Hb is the most abundant protein in human red blood cells and makes up 95% of the cell cytoplasm (Bryk and Wisniewski 2017). As a result, *Plasmodium* parasites have evolved a metabolic pathway that takes advantage of this abundant nutrient source; *Plasmodium falciparum*, for example, digests 60%–80% of the hemoglobin in its host cell (Krugliak et al. 2002; Sigala and Goldberg 2014). Hemoglobin digestion involves three main steps: the import of host hemoglobin, the breakdown of host hemoglobin into oligopeptides, and then the further breakdown of these oligopeptides into amino acids (Figure 1A).

The initial uptake of hemoglobin occurs in the first 18–30 h post red blood cell invasion and leads to the formation of a single large vacuole (Elliott et al. 2008), which is acidic in nature (pH 4.5–5.5) and becomes the site of hemoglobin digestion (Goldberg et al. 1990). The parasite continues to endocytose hemoglobin during the trophozoite and schizont stages via a cytostome‐mediated pathway (Elliott et al. 2008). Several parasitic proteins involved in hemoglobin digestion have been localized to the cytostome, including Kelch13 and other associated proteins, known as the Kelch13 compartment (Birnbaum et al. 2020; Schmidt et al. 2023).

Once inside the digestive vacuole, host hemoglobin is catabolized into oligopeptides, then dipeptides, and finally amino acids, which are incorporated into parasite protein synthesis. This is necessary for survival as malaria parasites are auxotrophic for most amino acids and must regulate their growth based on amino acid availability (Marreiros et al. 2023). The initial breakdown of hemoglobin to oligopeptides is completed by histo‐aspartic protease (plasmepsin III), falcipain 2, plasmepsin II, and plasmepsin IV (Chugh et al. 2013). These oligopeptides are then broken down to dipeptides and subsequently amino acids by falcilysin and multiple aminopeptidases (M1‐family alanyl aminopeptidase, M17‐family leucyl aminopeptidase, aminopeptidase p, dipeptidyl aminopeptidase 1, dipeptidyl aminopeptidase 3) (Eggleson et al. 1999; Klemba et al. 2004; Stack et al. 2007; Ragheb et al. 2011; Drinkwater et al. 2016).

A byproduct of hemoglobin digestion is the release of cytotoxic free heme, which contains ferrous iron that can produce reactive oxygen species that oxidize proteins, inhibit protein function, and disrupt membrane lipid bilayers (Sigala and Goldberg 2014). Many organisms including both animals and plants deal with free heme via the heme oxygenase pathway (Wilks 2002). *P. falciparum*, however, has a divergent heme oxygenase and lacks a canonical heme oxygenase pathway (Sigala and Goldberg 2014; Sigala et al. 2012). Instead, the parasite detoxifies heme by converting it to β‐hematin, a brown crystal also known as hemozoin. The iron remains bound to the heme molecules within hemozoin, restricting its oxidative potential. Heme detoxification protein (HDP) is hypothesized to be involved in hemozoin formation: as host hemoglobin is initially degraded by endopeptidases into oligopeptides and heme, HDP binds the exposed heme and positions it for dimer formation (Chugh et al. 2013; Nakatani et al. 2014). The heme dimers are then released from HDP and form hemozoin crystals via hydrogen bonds, possibly by interacting with lipids in lipid nanospheres (Nakatani et al. 2014; Pagola et al. 2000; Jani et al. 2008).

Hemoglobin digestion is required for the function of the antimalarial prodrug artemisinin as its endoperoxide bridge must be cleaved to produce the active drug, which is catalyzed by the iron within free heme (Chugh et al. 2013; Klonis et al. 2011). The active drug dihydroartemisinin then causes parasite death by creating free radicals that undergo oxidative reactions and non‐specifically damage parasite proteins and membranes (Hartwig et al. 2009). Artemisinin resistance is defined as a reduced clearance of parasites due to a decrease in drug susceptibility (Witkowski et al. 2013; Saralamba et al. 2011; Dondorp et al. 2009) and is associated with point mutations in the Kelch13 protein (the most prevalent of which is Kelch13^C580Y^) (Ariey et al. 2014; Straimer et al. 2015; Miotto et al. 2015). The Kelch13^C580Y^ mutation reduces the rate of hemoglobin endocytosis in ring stage parasites, and therefore reduces the rate of artemisinin activation (Birnbaum et al. 2020). This confers partial artemisinin resistance (Birnbaum et al. 2020) and correlates with artemisinin resistance in the field (Witkowski et al. 2013; Saralamba et al. 2011; Dondorp et al. 2009). A broad understanding of hemoglobin digestion is essential for our collective grasp of parasite biology and drug resistance, yet our study of this pathway has been restricted to solely mammalian‐infective *Plasmodium* thus far.

In contrast to the five species of *Plasmodium* parasites that can infect humans, over 100 described species of *Plasmodium* use lizards as their vertebrate hosts around the world (Telford Jr. 2008). As mammalian *Plasmodium* likely diverged from a sauropsid (bird, lizard) haemosporidian ancestor (Galen et al. 2018; Waters et al. 1991), sampling from this diverse group of parasites will help accurately reconstruct the evolution of traits in both mammalian and non‐mammalian malaria (Galen et al. 2018). In fact, genomic studies of bird‐infective taxa have already identified shared genes with human malaria parasite relatives (e.g., Videvall et al. 2017). Broad taxon sampling across the *Plasmodium* phylogenetic tree is necessary for these comparative analyses to continue, however, the genomic diversity of lizard‐infective *Plasmodium* remains unexplored.

*Plasmodium floridense*, *Plasmodium azurophilum*, and *Plasmodium leucocytica* infect *Anolis* (anole) lizards throughout the Caribbean islands and the surrounding mainland (Telford Jr. 2008; Falk et al. 2015), including the *Anolis sabanus* lizard on Saba Island (Staats and Schall 1996; Perkins 2000; Schall and Staats 2002). A single lizard host can be infected with one, two, or even all three of these parasites due to their unique intracellular niche partitioning. Like other *Plasmodium* species, *P. floridense* infects red blood cells and produces hemozoin (Thompson and Huff 1944). Conversely, *P. azurophilum* infects red blood cells while not producing any detectable hemozoin, and *P*. *leucocytica* infects white blood cells (Perkins 2000), thereby not relying on hemoglobin metabolism at all. Because of these unique phenotypes, *P. azurophilum* and *P*. *leucocytica* have seemingly evolved alternatives to the typical hemoglobin digestion pathway for amino acid acquisition. Additionally, since non‐mammalian erythrocytes are nucleated and are transcriptionally active (Waits et al. 2020), they may present a very different environment to the *Plasmodium* parasites that inhabit them. As a result, we hypothesize that these parasites have either lost the genes involved in this pathway or have alternative uses for these genes outside of solely hemoglobin digestion, which may relate to cellular niche partitioning. Investigating this could provide insights into the biology of hemoglobin digestion across *Plasmodium* evolutionary history. To investigate this, we examine the proteins involved in this pathway in these three *Plasmodium* species via transcriptomic and selection analyses. As there is currently no whole genome sequence available for any lizard‐infective *Plasmodium* species, this is the first evidence for the conservation of these genes across the *Plasmodium* tree. We argue that these data can be useful for the broader apicomplexan field as the transcriptomes presented in this paper can be used comparatively and mined for future transcripts of interest.

## 2 Results

### 2.1 Stringent Filtering Results in Higher Quality Parasite Transcriptome Assemblies From Host‐Contaminated Field Samples

A meta‐transcriptome (Table 1) was assembled *de novo* from field isolated lizard blood samples and contains 248,849 contigs (without isoforms) out of 120,323,340 assembled bases. The average contig length is 446.94 base pairs (bp) with a N50 value of 451 bp. This meta‐transcriptome is unfiltered and contains reads from all three *Plasmodium* species as well as the *Anolis* host. This is reflected by the GC content (42.28%), which matches the host rather than the *Plasmodium* species. Three parasite meta‐transcriptomes (Table 1) were created using reads from just the samples that contained only one of the respective species (Table S1). These transcriptomes were also created de novo; however, they were filtered using ContamFinder (Borner and Burmester 2017) to retain known *Plasmodium* contigs only. Due to this strict filtering, the total number of contigs (without isoforms) in these three assemblies range from 2744 to 2791 from ~2.5 million assembled bases, which is lower than published avian *Plasmodium* transcriptomes (Videvall et al. 2017; Weinberg et al. 2019; Lauron et al. 2015). The average contig length and N50 are significantly better in the filtered parasite assemblies versus the unfiltered meta‐assembly. The average contig lengths range from 870.76 to 878.39, and the N50 values range from 993 to 1002, which is comparable to published avian malaria assemblies (Videvall et al. 2017; Weinberg et al. 2019; Lauron et al. 2015). Lastly, the filtered assemblies have a GC content that matches an expected GC content for *Plasmodium* parasites. Unsurprisingly, due to their evolutionary relatedness, the two sister species have a similar GC content (25.16% for *P. azurophilum* and 25.30% for *P. leucocytica*), while *P. floridense* has a lower GC content at 22.50%. The GC content for *P. azurophilum* and *P. leucocytica* is higher than other published *Plasmodium* transcriptomes, which typically fall around 21%–22% (Videvall et al. 2017; Weinberg et al. 2019), however, the avian malaria transcriptome for *Plasmodium delichoni* also has a higher reported GC content of 23.93% (Weinberg et al. 2019).

**TABLE 1:** Transcriptome summary statistics.

Transcriptome	Total contigs (without isoforms)	Average contig length (bp)	Contig N50 value (bp)	GC content (%)	Total assembled bases
Unfiltered meta‐transcriptome	248,849	446.94	451	42.28	120,323,340
*P. floridense* meta‐transcriptome	2791	870.76	993	22.50	2,467,729
*P. azurophilum* meta‐transcriptome	2744	878.39	1002	25.16	2,489,368
*P. leucocytica* meta‐transcriptome	2763	876.48	1000	25.30	2,483,958

*Note:* The meta‐transcriptome was constructed *de novo* using all samples and was not filtered to remove host read contaminants. *Plasmodium* species meta‐transcriptomes include reads from multiple samples but only those correlating to each respective species. *Plasmodium* species meta‐transcriptomes were filtered using ContamFinder (Borner and Burmester 2017) to remove host reads. Transcriptome summary statistics were calculated using Trinity_Stats.pl in Trinity (Haas et al. 2013).

**FIGURE 2:**
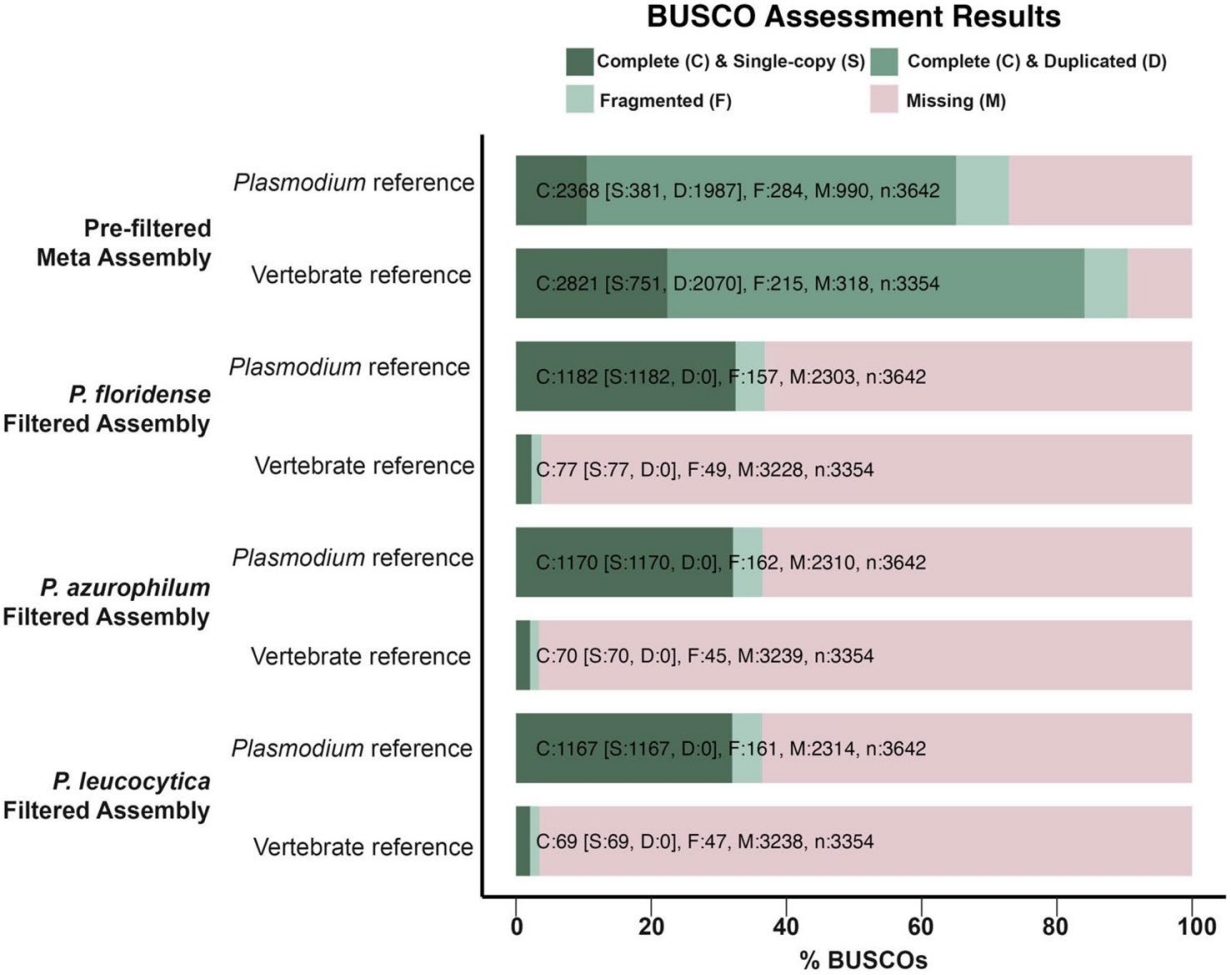
BUSCO assessment of parasite transcriptome assemblies. BUSCO (Manni, Berkeley, Seppey, and Zdobnov 2021) graph indicates completeness of the parasite transcriptome assemblies based on expected gene counts using a *Plasmodium* reference (plasmodium_odb10 lineage from the OrthoDB database; Zdobnov et al. 2021). The BUSCO test was also run using the vertebrata_odb10 lineage (Zdobnov et al. 2021) to assess lizard host read contamination. Both assessments were run for the prefiltered meta‐assembly as well as the ContamFinder filtered assemblies (*Plasmodium floridense* only meta‐assembly, *Plasmodium azurophilum* only meta‐assembly, and *Plasmodium leucocytica* only meta‐assembly). The % complete & single‐copy, complete & duplicated, fragmented, and missing reads are indicated by color on the bar graph. The numbers for each group are also listed on top of the bar graph (*n* value = total number of BUSCOs in given reference). Plot was created in ggplot2 in R (Wickham 2016).

BUSCO (Manni, Berkeley, Seppey, and Zdobnov 2021; Manni, Berkeley, Seppey, Simao, and Zdobnov 2021) analysis (Figure 2) was performed on the unfiltered and filtered meta‐transcriptomes to assess the completeness of the assemblies relative to a database of universal single‐copy orthologs (Zdobnov et al. 2021). Each assembly was evaluated against a *Plasmodium* reference database (plasmodium_odb10) (Zdobnov et al. 2021) to assess parasite transcriptome completeness and a vertebrate reference database (vertebrata_odb10) (Zdobnov et al. 2021) to assess host read content. A high‐quality assembly is expected to have a high percentage of single‐copy genes and a low duplication score (Manni, Berkeley, Seppey, and Zdobnov 2021). The unfiltered meta‐assembly is, therefore, of low quality and has a high proportion of contaminating host contigs. The filtered meta‐assemblies for each species have an increase in quality (higher proportion of *Plasmodium* single‐copy genes and 0% duplication score), but still are missing data as they only have ~30% completeness compared to the *Plasmodium* database. This is likely due to the nature of the field samples (host read contaminated, small sample volume). The filtered meta‐assemblies also have a small proportion of vertebrate single copy genes (~2% of the vertebrate database). A BLAST analysis was performed using the transcripts and the parasite and vertebrate databases, and a proportion of the transcripts align to both databases. The small proportion of potential vertebrate transcripts in the final parasite assemblies are likely due to this overlap. Overall, the host contamination is greatly reduced in the final parasite assemblies after the filtering steps.

### 2.2 Transcriptomes Provide More Insight for Sample Characterization

Transcript expression counts were calculated for every lizard sample by mapping the sample reads back to the three filtered parasite meta‐assemblies. This allowed for characterization of each lizard sample. The transcriptome sequence data indicate that some lizard blood samples had *Plasmodium* infections that were not previously detected by eye (Table S1). Additionally, the transcriptomic data show that 9/14 infected lizards had coinfections rather than one infection (Table S1). Parasitemia (the number of parasites in the blood, calculated by # parasite‐infected cells/10,000 red blood cells) is low for these red blood cell parasite infections as most samples had < 2% infected cells (Figure S1). Samples as low as 0.06% parasitemia were detected via microscopy; however, infections likely exist below this parasitemia level and were undetectable by eye (Table S1). For example, samples 8 and 12 were diagnosed as uninfected prior to RNA sequencing, but have parasite reads and were diagnosed as infected post sequencing (Table S1). These two samples were not used for further parasite gene analysis due to this diagnosis discrepancy.

**FIGURE 3:**
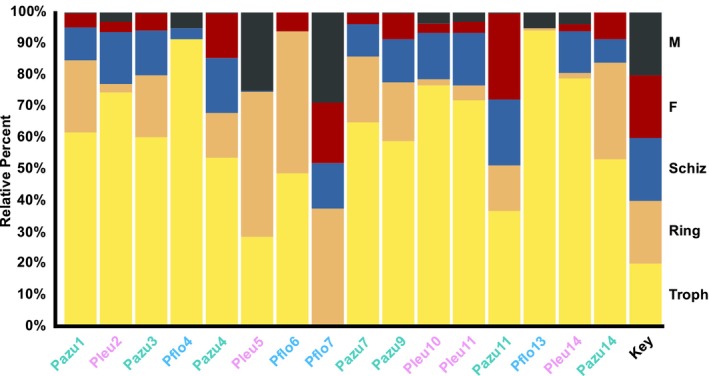
Predicted parasite life stage abundance within each sample using CIBERSORTx. The RNAseq samples include a mixture of parasite life stages and were not synchronized experimentally due to the limitations of field sampling methods. To better characterize which life stages make up each sample, a mathematical model was employed to deconvolute the bulk RNAseq data. Relative percent was calculated using CIBERSORTx (Newman et al. 2019) and a published *Plasmodium* reference (Tebben et al. 2022). Parasite life stages are labeled as follows: Troph = trophozoite, ring = ring, schiz = schizont, F = female gametocyte, M = male gametocyte. Due to the use of a *P. falciparum* reference, rings are denoted here as a life stage, but this morphology is not seen in these three lizard‐infective parasites and this stage is referred to as early trophozoite throughout the rest of the manuscript. Samples are labeled with species name (Pflo = *P. floridense* , Pazu = *P. azurophilum*, Pleu = *P. leucocytica*) followed by sample number and color coordinated by species to match other figure legends. A stacked bar chart was produced in CIBERSORTx output.

Due to the nature of working with field samples, the parasites were not synchronized experimentally so these samples include a mixture of life stages. Microscopy was also not ideal for determining the proportion of life stages in each sample as life stages can be mischaracterized, misassigned in multi‐infections, or missed altogether due to low parasite abundance. In order to characterize the composition of parasite life stage within each sample and confirm the presence of hemoglobin‐digesting life cycle stages within each sample, a statistical model was employed. This mathematical model estimates cell composition in bulk RNAseq samples using reference gene expression datasets and is termed gene expression deconvolution. Deconvolution of samples was conducted in CIBERSORTx (Newman et al. 2019) using a published *Plasmodium* reference data set (Tebben et al. 2022) and output was charted as relative parasite life stage abundance (Figure 3). While the published *Plasmodium* reference data set utilizes gene profiles from *P. falciparum* 3D7 single cell data, the authors note that it is transferable to other *Plasmodium* species and can still reliably deconvolute bulk RNAseq data from related species (Tebben et al. 2022).

The estimated life stage abundances confirm that while the samples are mixed, the hemoglobin‐digesting life stage (trophozoite) is present in the samples at a high relative percentage (Figure 3). This data also indicates that the samples have all parasite life stages circulating in the blood (although at different proportions). While organs from infected lizards will need to be analyzed to confirm, these expression profiles imply that none of the parasite life stages sequester in other tissue types. Notably, we observed a species‐uniquely high proportion of female gametocytes in the *P. azurophilum*‐infected samples and relatively little to no male gametocytes (Figure 3). The difference in life stage abundance across samples could represent a true pattern; that life stage proportions (especially regarding gametocytes) vary across species (Schall 1996). Alternatively, gene expression at different life stages may not necessarily exhibit the same patterns across all *Plasmodium* species, which could impact these estimated abundances. Given these caveats, this method of characterizing field samples is not perfect; however, it still provides some insight as the samples can be analyzed relative to one another.

### 2.3 Overall Transcript Expression Varies by Parasite Species

**FIGURE 4:**
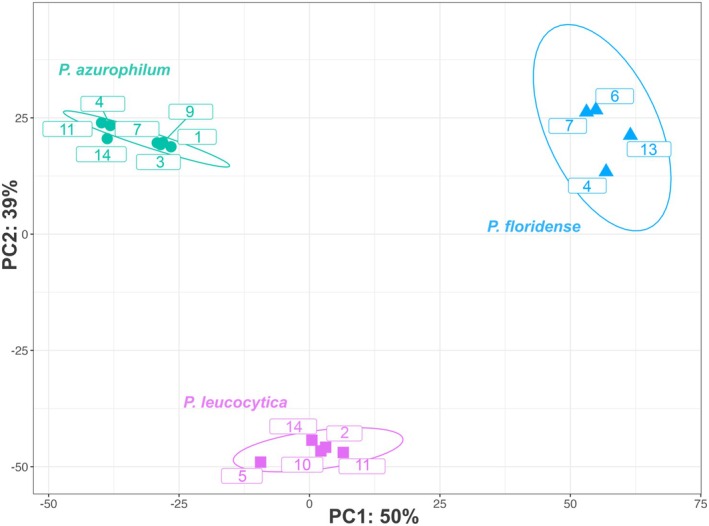
Principal component analysis of three lizard‐infective *Plasmodium* species transcriptome samples for top 500 transcripts. Bulk RNAseq was performed on *Anolis sabanus* blood infected with *Plasmodium floridense*, *Plasmodium azurophilum*, and *Plasmodium leucocytica*. 4 *P. floridense* , 7 *P. azurophilum*, and 5 *P. leucocytica* infections are represented from 12 lizard blood samples (lizard sample number is labeled on the PCA and correlates to sample number in Table S1). Samples cluster by *Plasmodium* species within a 95% confidence interval ellipse. The first two principal components account for 89% of variation. The PCA was constructed using the DESeq2 and ggplot2 packages in R (Love et al. 2014; Wickham 2016).

Principal component analysis (PCA) was used to characterize the samples overall. For the top 500 most differentially expressed genes, samples cluster by *Plasmodium* species (Figure 4). This indicates that transcription is most similar within *Plasmodium* species type, despite variation in other factors, such as parasitemia or the proportion of life stages in each sample. Whether or not parasites exist as a single infection or a multi‐infection did not appear to influence within‐species variation; however, more data would be necessary to robustly determine this (Figure S2). The first two principal components account for most of the variation in the data (Figure S3). The first principal component separates out the two sister species (*P. azurophilum* and *P. leucocytica*) from *P. floridense* , while the second principal component groups the two red blood cell inhabiting species (*P. azurophilum* and *P. floridense* ) together away from *P. leucocytica* (white blood cell inhabiting) (Figure 4). The top 5 transcripts driving the differentiation are mapped onto a PCA (Figure S4) and labeled with the gene ID for the orthologous gene in *Plasmodium gallinaceum*. PC1 is driven by a signal peptide peptidase (PlasmoDB Gene ID: PGAL8A_00216300), U4/U6.U5 tri‐snRNP‐associated protein 1 (PGAL8A_00392600), WD repeat‐containing protein (PGAL8A_00227400), and two conserved proteins of unknown function (PGAL8A_00408300, PGAL8A_00504500). PC2 is driven most by N‐acetylglucosaminyl‐phosphatidylinositol de‐N‐acetylase (PGAL8A_00153200), selenide water dikinase (PGAL8A_00423200), adenylyltransferase and sulfurtransferase UBA4 (PGAL8A_00171900), U3 small nucleolar RNA‐interacting protein 2 (PGAL8A_00127800), and one conserved protein of unknown function (PGAL8A_00517000). No distinct pattern can be reported from this group of proteins that is contributing to the variation in PC1 and PC2. For example, when gene ontology (GO) terms were analyzed for each gene via PlasmoDB (The Plasmodium Genome Database Collaborative 2001), there were no repeating or common GO terms.

### 2.4 Lizard‐Infective *Plasmodium* Parasites Have and Express Orthologs for Hemoglobin Digestion Genes

The *P. floridense* , *P. azurophilum*, and *P. leucocytica* meta‐transcriptomes were analyzed for orthologous sequences of known hemoglobin digestion genes. Orthologs were detected for 10 out of 16 analyzed genes in all three species (Table 2). Orthologous transcripts are present for each step in the hemoglobin digestion pathway (Figure 1B,C). No ortholog was detected for two of the genes involved in hemoglobin uptake, namely ubiquitin carboxyl‐terminal hydrolase 1 (UBP1) and protein KIC7 (KIC7). KIC7 also does not have an ortholog in bird‐infecting *Plasmodium* (Table 2) and, therefore, may not be present in the sauropsid *Plasmodium* clade at all. Only the Kelch13 compartment proteins found to impair endocytosis when inactivated (Birnbaum et al. 2020) were analyzed, so there are more proteins involved in the endocytosis machinery (Birnbaum et al. 2020; Schmidt et al. 2023) to be analyzed in the future. Additionally, no transcript was found for four digestive enzymes in the lizard parasites: cysteine proteinase falcipain 2a (FP2A), cysteine proteinase falcipain 2b (FP2B), plasmepsin II (PMII), and histo aspartic protease/plasmepsin III (PMIII) (although orthologs were detected for plasmepsin IV). Targeted RNA/DNA sequencing for detection of these genes would confirm whether they are truly absent from the genomes or not. We attempted to do so via conventional primer design and PCR based on sequences from related species; however, we were not able to amplify the intended genes. A targeted sequencing approach, such as selective whole genome amplification, may be more successful. The three species did have an ortholog for falcipain 1, which was confirmed using a phylogenetic tree analysis—the sequences grouped with sequences for falcipain 1 from other species (rather than falcipain 2a/b) (Figure S5). The same analysis was performed for an additional plasmepsin transcript (different from the PIV ortholog), which grouped with plasmepsin VI orthologs rather than plasmepsin II or III (Figure S5) and is thus unlikely to play a role in hemoglobin digestion as plasmepsin VI is implicated in transmission‐stage parasites (Ecker et al. 2008; Nasamu et al. 2020).

**TABLE 2:** Presence/absence of sixteen genes known to be involved in the *Plasmodium* hemoglobin digestion pathway.

Gene	Step in Hb digestion pathway	Present in *P. floridense*	Present in *P. azurophilum*	Present in *P. leucocytica*	Present in *P. gallinaceum*	Potential *Babesia* species ortholog	Potential *Theileria* species ortholog	Potential *Toxoplasma* species ortholog	Essential in *P. falciparum*	Essential in *P. berghei*
Kelch13 (K13)	Hb Import	Y	Y	Y	Y	Y	Y	Y	Y	n.d.
Ubiquitin Carboxyl‐terminal Hydrolase 1 (UBP1)	Hb Import	N	N	N	Y	Y	Y	Y	Y	n.d.
Ap2 Complex Subunit μ (Ap2‐μ)	Hb Import	Y	Y	Y	Y	N	N	Y	Y	n.d.
KIC7 (KIC7)	Hb Import	N	N	N	N	N	Y	Y	Y	n.d.
EPS15‐like Protein (EPS15)	Hb Import	Y	Y	Y	Y	N	N	Y	Y	n.d.
Cysteine Proteinase Falcipain 2a (FP2A)	Hb Digestion	N	N	N	Y	Y	Y	Y	N	N
Cysteine Proteinase Falcipain 2b (FP2B)	Hb Digestion	N	N	N	Same possible orthologs as FP2a	Same possible orthologs as FP2a	Same possible orthologs as FP2a	Same possible orthologs as FP2a	N	n.d.
Plasmepsin II (PMII)	Hb Digestion	N	N	N	N	Y	N	Y	N	n.d.
Histo Aspartic Protease/Plasmpesin III (PMIII)	Hb Digestion	N	N	N	N	N	N	N	Y	n.d.
Plasmepsin IV (PM4)	Hb Digestion	Y	Y	Y	Y	Y	N	Y	N	N
Falcilysin (FLN)	Hb Digestion	Y	Y	Y	Y	Y	Y	Y	Y	Y
M1‐family alanyl aminopeptidase (M1AAP)	Hb Digestion	Y	Y	Y	Y	Y	Y	Y	Y	Y
M17 leucyl aminopeptidase (LAP)	Hb Digestion	Y	Y	Y	Y	Y	Y	Y	Y	N
Aminopeptidase P (APP)	Hb Digestion	Y	Y	Y	Y	Y	N	Y	Y	N
Dipeptidyl Aminopeptidase 1 (DPAP1)	Hb Digestion	Y	Y	Y	Y	Y	Y	Y	Y	N
Heme Detoxification Protein (HDP)	Hz Formation	Y	Y	Y	Y	Y	Y	N	Y	N

*Note:* Orthologous sequences for the genes detected in the lizard malaria transcriptomes (green, Y) or not (white, N). Presence/absence of orthologous sequences in *Plasmodium gallinaceum*, *Babesia bigemina*, *Theileria parva*, and *Toxoplasma gondii* is listed. Orthologous sequences for *P. gallinaceum* were searched in PlasmoDB (The Plasmodium Genome Database Collaborative 2001). Potential *Babesia*, *Theileria*, and *Toxoplasma* species orthologs were determined by searching their reference proteomes for a protein match with DIAMOND (Buchfink et al. 2021). Whether or not the gene has been published to be essential in *Plasmodium falciparum* and *Plasmodium berghei* is also listed (n.d. = no data) (Birnbaum et al. 2020; Lin et al. 2015).

Interestingly, potential orthologous sequences were also identified in three other apicomplexan parasite species (Table 2; Table S2). *Babesia* parasites invade red blood cells but do not produce hemozoin, *Theileria* parasites invade white blood cells, and *Toxoplasma* parasites are generalists that can invade nearly any nucleated cell and do not produce hemozoin. BLAST analysis pulled out potential orthologous proteins for 12 of these genes in *Babesia bigemina*, 10 in *Theileria parva*, and 14 in *Toxoplasma gondii* (Table 2; Table S2). The possible presence of these proteins in distantly related apicomplexan parasites that have similar phenotypes to two of the lizard‐infective malaria parasites (no hemozoin formation), suggest other potential functions for these proteins. Further analyses (protein structure prediction and protein functional analyses) will be necessary to determine if these proteins are true orthologs, especially because some came up with multiple possible blast hits (Table S2).

**FIGURE 5:**
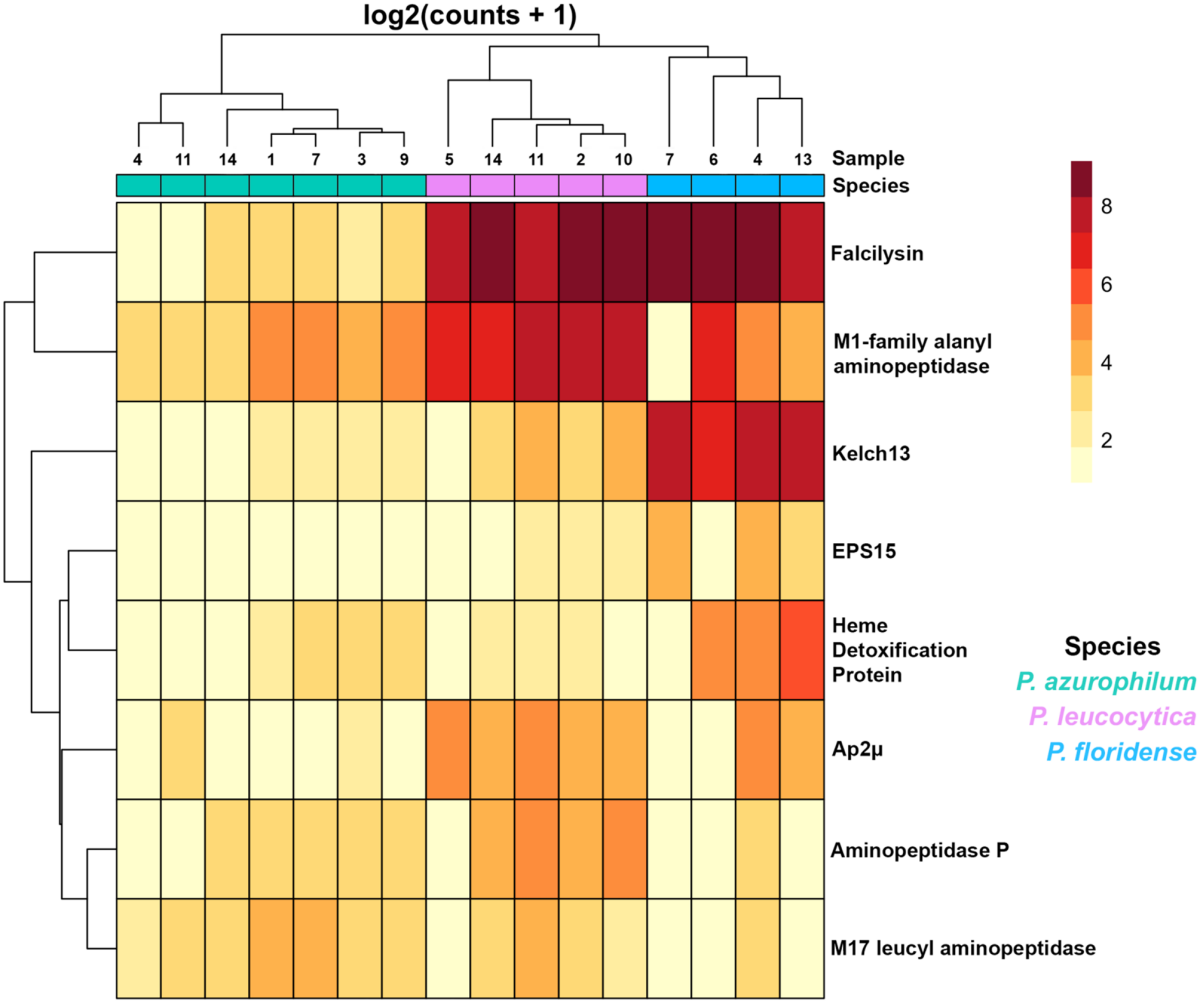
Expression for known genes in the hemoglobin uptake and digestion pathway. Heatmap depicting expression level (log_2_(counts+1)) for genes known to be involved in the hemoglobin uptake and digestion pathway in mammalian *Plasmodium*. Only genes expressed by the lizard‐infective parasites (with a cutoff value of 1 TPM) are represented in this heatmap. Each column corresponds to a transcriptome sample and is labeled with the sample number and species. Dendrogram indicates clustering by expression. Heatmap constructed using the DESeq2 package in R (Love et al. 2014).

Transcripts for the hemoglobin digestion genes with an expression value of > 1TPM are displayed in a heatmap for all samples (Figure 5). Eight out of the 10 identified genes had an expression value above the cutoff and are shown on the heatmap. Samples (columns) are clustered by expression profiles and group together by species, following the trend shown in the PCA (Figure 4). Similarly, genes (rows) are clustered and arranged by expression. Using these data, *P. floridense* expresses kelch13 and heme detoxification protein at the highest levels, and *P. leucocytica* expresses M1‐family alanyl aminopeptidase at the highest levels. *P. floridense* and *P. leucocytica* express falcilysin at similar. Levels *P. azurophilum* appears to express all transcripts at the lowest levels (Figure 5). The expression levels of these genes should be further validated to determine if this trend is consistent.

### 2.5 Shifts in Selection on Hemoglobin Digestion Genes

Due to the condition and small sample size of the field samples, an analysis of selection pressure was used as a proxy for protein functionality instead of in vitro protein analysis. A new ecological niche can remove functional constraints and result in a decrease in selective pressure (termed relaxed selection). Relaxed selection has been detected in a range of species (Wertheim et al. 2015), including trichomonad parasite species in response to a new host environment (host switch) (Sullivan et al. 2024). Alternatively, selection can increase with the introduction of new functional constraints (termed intensification) (Wertheim et al. 2015). A gene with signatures of relaxed selection could indicate shifts in the functional importance of that gene are occurring and/or, in the extreme scenario, the development of that gene into a pseudogene in the future. In contrast, a gene under selection intensification could indicate stronger purifying or positive selection is acting upon that gene. Because *P. azurophilum* and *P. leucocytica* no longer produce hemozoin (and *P. leucocytica* inhabits an entirely new cellular niche), we hypothesize that there will be signatures of relaxed selection on hemoglobin uptake and digestion genes relative to other *Plasmodium* species.

Multiple sequence alignments and gene trees (Figure S6) were constructed for all expressed hemoglobin digestion genes. Orthologous sequences were accessed from PlasmoDB (The Plasmodium Genome Database Collaborative 2001) (Table S3). The data were imported into HyPhy (Weaver et al. 2018) and relaxed selection was tested for each gene using RELAX (Wertheim et al. 2015). RELAX was used to detect relative changes in selective pressures in the lizard‐infective malaria genes using dN/dS ratios. This test is relative, meaning the signature of intensification or relaxation along the branches of interest is in comparison to selected reference branches along the phylogenetic tree (Wertheim et al. 2015). RELAX was used to detect changes in selection on the genes for the two parasites that do not produce hemozoin (*P. azurophilum* and *P. leucocytica*) relative to two reference groups: (1) all the *Plasmodium* species in the dataset (entire tree) and (2) *Plasmodium* species only in the sauropsid (bird, lizard) clade (sauropsid clade only) (Table 3). As a comparison, the test was also run on the *P. floridense* genes compared to the entire tree and the sauropsid clade, as well as on the genes in all the mammalian *Plasmodium* species compared to the sauropsid clade (Table 3). Because the mammalian parasites produce hemozoin and digest hemoglobin, we hypothesize that there would not be any signatures of relaxed selection in these genes.

**TABLE 3:** Relaxed selection analysis for the genes involved in hemoglobin uptake and digestion.

Gene	*P. azurophilum* & *P. leucocytica*		*P. leucocytica* only	*P. azurophilum* only	*P. floridense*		All mammalian‐infective species	Test
	Entire tree	Sauropsid clade only	Entire tree (without *P. azurophilum*)	Entire tree (without *P. leucocytica*)	Entire tree (without *P. azurophilum* & *P. leucocytica*)	Sauropsid clade only (without *P. azurophilum* & *P. leucocytica*)	Sauropsid clade only (without *P. azurophilum* & *P. leucocytica*)	References
Kelch13 (K13)	Relaxation (*K* = 0.61, *p* = 0.017)	Relaxation (*K* = 0.74, *p* = 0.208)	Relaxation (*K* = 0.17, *p* = 0.006)	Relaxation (*K* = 0.42, *p* = 0.009)	Relaxation (*K* = 0.27, *p* = 0.000)	Relaxation (*K* = 0.28, *p* = 0.000)	Intensification (*K* = 1.96, *p* = 0.000)	
Ap2 Complex Subunit μ (Ap2μ)	Relaxation (*K* = 0.08, *p* = 0.000)	Relaxation (*K* = 0.21, *p* = 0.000)	Relaxation (*K* = 0.02, *p* = 0.000)	Relaxation (*K* = 0.00, *p* = 0.005)	Relaxation (*K* = 0.00, *p* = 0.000)	Relaxation (*K* = 0.00, *p* = 0.000)	Intensification (*K* = 3.85, *p* = 0.000)	
EPS15‐like Protein (EPS15)	Relaxation (*K* = 0.44, *p* = 0.004)	Relaxation (*K* = 0.11, *p* = 0.000)	Relaxation (*K* = 0.00, *p* = 0.000)	Relaxation (*K* = 0.77, *p* = 0.070)	Relaxation (*K* = 0.97, *p* = 0.871)	Relaxation (*K* = 0.97, *p* = 0.880)	Relaxation (*K* = 0.88, *p* = 0.196)	
Dipeptidyl Aminopeptidase 1 (DPAP1)	Intensification (*K* = 4.47, *p* = 0.005)	Intensification (*K* = 1.04, *p* = 0.954)	Relaxation (*K* = 0.26, *p* = 0.184)	Intensification (*K* = 2.80, *p* = 0.010)	Intensification (*K* = 5.59, *p* = 0.004)	Intensification (*K* = 2.20, *p* = 0.454)	Intensification (*K* = 13.25, *p* = 0.037)	
Plasmepsin IV (PM4)	Intensification (*K* = 6.16, *p* = 0.000)	Intensification (*K* = 4.35, *p* = 0.006)	Intensification (*K* = 10.49, *p* = 0.000)	Relaxation (*K* = 0.00, *p* = 0.000)	Relaxation (*K* = 0.00, *p* = 0.000)	Relaxation (*K* = 0.07, *p* = 0.078)	Intensification (*K* = 21.21, *p* = 0.000)	
Heme Detoxification Protein (HDP)	Intensification (*K* = 1.23, *p* = 0.333)	Intensification (*K* = 1.20, *p* = 0.513)	Intensification (*K* = 1.24, *p* = 0.521)	Intensification (*K* = 1.40, *p* = 0.365)	Relaxation (*K* = 0.55, *p* = 0.171)	Relaxation (*K* = 0.72, *p* = 0.348)	Relaxation (*K* = 0.88, *p* = 0.553)	
Falcilysin (FLN)	Relaxation (*K* = 0.24, *p* = 0.005)	Relaxation (*K* = 0.71, *p* = 0.637)	Relaxation (*K* = 0.00, *p* = 0.000)	Relaxation (*K* = 0.00, *p* = 0.002)	Relaxation (*K* = 0.00, *p* = 0.000)	Relaxation (*K* = 0.00, *p* = 0.000)	Intensification (*K* = 5.63, *p* = 0.000)	
M1‐family alanyl aminopeptidase (M1AAP)	Relaxation (*K* = 0.00, *p* = 0.000)	Relaxation (*K* = 0.00, *p* = 0.000)	Relaxation (*K* = 0.00, *p* = 0.000)	Relaxation (*K* = 0.00, *p* = 0.000)	Relaxation (*K* = 0.00, *p* = 0.000)	Relaxation (*K* = 0.10, *p* = 0.050)	Intensification (*K* = 13.64, *p* = 0.000)	
M17 leucyl aminopeptidase (LAP)	Relaxation (*K* = 0.87, *p* = 0.454)	Relaxation (*K* = 0.90, *p* = 0.625)	Relaxation (*K* = 0.79, *p* = 0.558)	Relaxation (*K* = 0.93, *p* = 0.772)	Relaxation (*K* = 0.78, *p* = 0.260)	Relaxation (*K* = 0.78, *p* = 0.324)	Intensification (*K* = 1.07, *p* = 0.755)	
Aminopeptidase P (APP)	Relaxation (*K* = 0.00, *p* = 0.000)	Relaxation (*K* = 0.18, *p* = 0.000)	Relaxation (*K* = 0.49, *p* = 0.008)	Relaxation (*K* = 0.04, *p* = 0.000)	Relaxation (*K* = 0.00, *p* = 0.000)	Relaxation (*K* = 0.00, *p* = 0.000)	Intensification (*K* = 6.08, *p* = 0.000)	

*Note:* RELAX (Wertheim et al. 2015) was run for *Plasmodium azurophilum* and *Plasmodium leucocytica* (combined and separate) relative to the entire *Plasmodium* gene tree (minus the outgroup) or the sauropsid clade only. As a comparison, the test was also run for *Plasmodium floridense* relative to the entire tree (minus the outgroup, *P. azurophilum*, and *P. leucocytica*) or the sauropsid clade only (minus *P. azurophilum*, and *P. leucocytica*). Selection was also measured for the entire mammalian clade relative to the sauropsid clade (minus *P. azurophilum*, and *P. leucocytica*). Results are listed and labeled by nonsignificant change (white), significant signatures of relaxed selection (green), and significant signatures of intensification (blue). Output from RELAX includes an estimated selection intensity (*K* value, *K* > 1 = intensified selection, *K* < 1 = relaxed selection) and a *p* value.

Six genes show significant signatures of relaxed selection when measured in *P. azurophilum* and *P. leucocytica* together relative to other *Plasmodium* species (Table 3): Kelch13, Ap2 complex subunit μ, EPS15‐like protein, falcilysin, M1‐family alanyl aminopeptidase, and aminopeptidase p. In contrast, heme detoxification protein and M17 leucyl aminopeptidase had no significant changes in selection, and dipeptidyl aminopeptidase 1 and plasmepsin IV had significant signatures of intensification (Table 3), which was not expected. Interestingly, when selection was analyzed separately for *P. azurophilum* and *P. leucocytica*, some genes show different selection intensities in each sister species (Table 3). When analyzed together, EPS15‐like protein shows signatures of relaxed selection; however, only the *P. leucocytica* transcript appears to be driving this, as no significant changes in selection were detected in the *P. azurophilum*‐only test. Similarly, dipeptidyl aminopeptidase 1 only has intensification in *P. azurophilum*, and plasmepsin IV only has intensification in *P. leucocytica*. Unexpectedly, significant relaxed selection was also detected in *P. floridense* for multiple genes: Kelch13, Ap2 complex subunit μ, plasmepsin IV, falcilysin, M1‐family alanyl aminopeptidase, and aminopeptidase p. These shifts in selection seen in *P. floridense* match those in *P. azurophilum* rather than other hemoglobin‐digesting parasites (Table 3). As hypothesized, the mammal control had no signatures of relaxed selection and either had no change in selective pressure or an intensification of selective pressure (Table 3).

### 2.6 Genes Not Under Relaxed Selection Could Have Alternative Functions in the Cell

Perhaps of particular interest are the genes that do not show any relative shifts in selection for the lizard malaria parasites, which include heme detoxification protein and M17 leucyl aminopeptidase. As mentioned, we hypothesize that the digestive enzymes are acting on other proteins and are therefore still necessary for parasite survival. Of these two proteins, heme detoxification protein is the only gene that has solely been implicated in hemoglobin digestion thus far (hemozoin formation). As *P. azurophilum* and *P. leucocytica* do not produce hemozoin (but transcribe HDP and have maintained selective pressure for HDP), we hypothesize that this gene has another function outside of hemozoin formation. An ortholog is also present in *Babesia* and *Theileria* parasites (Table 2; Table S2), which do not produce hemozoin, thereby adding support for an alternative function.

**FIGURE 6:**
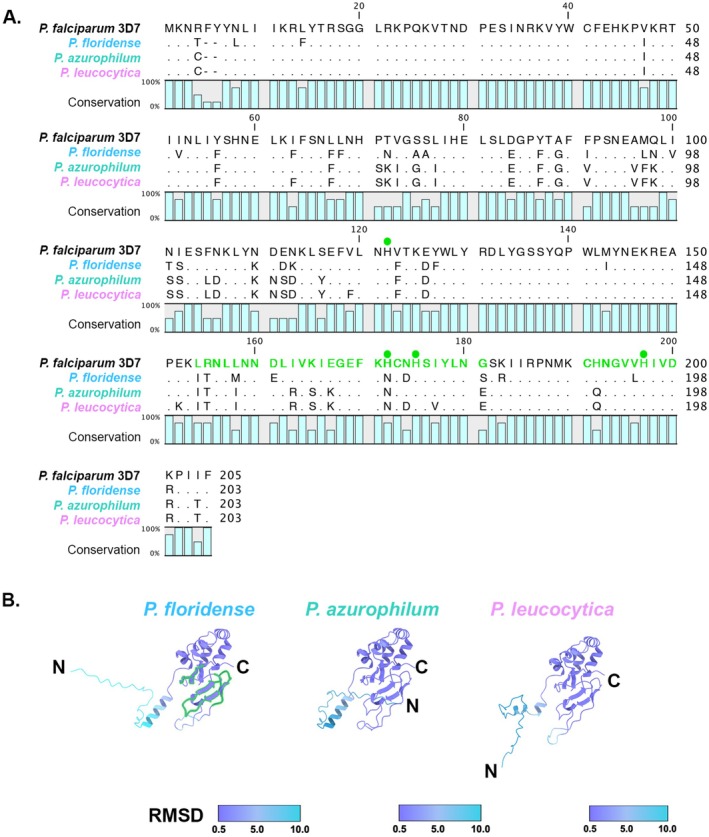
Multiple sequence alignment and protein structure prediction for heme detoxification protein. (A) Multiple sequence alignments were constructed in MUSCLE (Edgar 2004) for the coding sequence of each gene involved in the hemoglobin uptake and digestion pathway. The MSA for the predicted protein sequence for heme detoxification protein (HDP) is shown here (for ease of viewing, only the three lizard malaria sequences and *Plasmodium falciparum* sequence are shown). The graphic of this MSA was obtained via CLC Sequence Viewer v.8.0. Black dots represent conserved amino acids across the MSA relative to the *P. falciparum* 3D7 HDP sequence. The area highlighted in green is the predicted active site and green dots indicate histidine amino acids that may be involved in heme binding (Nakatani et al. 2014). (B) Predicted structures for *Plasmodium floridense*, *Plasmodium azurophilum*, and *Plasmodium leucocytica* heme detoxification protein. Protein structures were predicted using ColabFold v.1.5.2 (Mirdita et al. 2022). Purple‐blue gradient indicates root mean square deviation (RMSD) in the Cα positions was calculated for each best fit model relative to the predicted protein structure for *P. falciparum* 3D7 heme detoxification protein (obtained from AlphaFold; Jumper et al. 2021) using ChimeraX‐1.6.1 (Goddard et al. 2018). Green outlining on the *P. floridense* structure indicates the predicted binding region that is displayed in the MSA. N and C termini are labeled on the figures.

Protein analysis will also be necessary to determine whether this gene is still functional in the lizard parasites. Experimental protein expression, for example, would be beneficial; however, computational protein prediction can also act as an initial analysis (Figure 6). The protein structures for heme detoxification protein from *P. floridense* , *P. azurophilum*, and *P. leucocytica* were predicted in ColabFold and mapped onto the predicted *P. falciparum* protein structure (Figure 6B). Predicted structures for all three lizard malaria parasites align closely to the predicted *P. falciparum* structure in the ordered regions of the protein (Figure 6B). While protein structure is relatively conserved, there are a few differences in the amino acid composition. One histidine originally predicted to be a heme binding site in the *P. falciparum* HDP is not conserved in *P. floridense* , *P. azurophilum*, and *P. leucocytica* (His172Asn) (Figure 6A). Recent structure and binding prediction data, however, indicate that this histidine in addition to His175 is not necessary for binding heme after all (Klar et al. 2024). Our sequence data adds support for this new binding prediction as we hypothesize that the heme binding sites should be conserved across all the hemozoin‐producing *Plasmodium* parasites. Because *P. floridense* lacks His172 but still produces hemozoin, this would indicate that His172 is not necessary for heme binding. Based on the protein structure prediction data, we expect the heme detoxification protein in all three lizard malaria parasites to be functional. Combined with the other evidence already discussed, we predict that HDP has another function outside of hemozoin formation in *P. azurophilum* and *P. leucocytica* (and perhaps the other *Plasmodium* parasites as well).

## 3 Discussion

### 3.1 Hemoglobin Digestion and Hemozoin Formation Is a Conserved Process Across the *Plasmodium* Phylogenetic Tree

10/16 genes in the hemoglobin digestion pathway are present in all three lizard‐infective *Plasmodium* samples (Table 2). As there are no whole genome sequences available for these parasites, this is the first evidence that these genes exist in the lizard‐infective malaria genomes and are conserved across the *Plasmodium* phylogenetic tree. At baseline, this tells us that hemoglobin digestion genes exist even in parasites that one would not expect if these genes only play a role in hemoglobin digestion (thereby implying alternate functions). The six genes that were not identified are either not conserved in these parasites, or they do exist in the genome, but are not present in the meta‐transcriptomes (due to missing data). Our transcriptomes only cover ~30% of the *Plasmodium* transcriptome (Figure 2), so missing data is likely to play a large role in this observation. Given the evolutionary relatedness between bird and lizard‐infective malaria species, we hypothesize the latter is the case for all the genes that have an ortholog in the bird‐infective *Plasmodium* species (Table 2).

The expression patterns of these genes indicate higher expression for genes involved in hemoglobin uptake (Kelch13, Eps15) as well as hemozoin formation (HDP) in the hemozoin forming parasite ( *P. floridense* ) compared to the parasites that do not produce hemozoin (*P. azurophilum* and *P. leucocytica*). As discussed, the samples include a mixture of life stages, which could be playing a role in these expression levels. If the predicted life stage abundances are taken into consideration (Figure 3), however, all samples have relatively high proportions of trophozoites regardless of species. If all species are utilizing these transcripts at this stage, we would not expect one species to have higher relative expression for these genes versus another.

### 3.2 Shifts in Selection May Reflect Adaptations to the Lizard Host Cellular Environment

Genes involved in hemoglobin uptake (Kelch13 and Ap2μ) are under relaxed selection in both *P. azurophilum* and *P. leucocytica*. Interestingly, EPS15, which is also involved in hemoglobin uptake, is only under relaxed selection in *P. leucocytica*. Some of the digestive enzymes (falcilysin, M1‐family alanyl aminopeptidase, aminopeptidase p) show significant signatures of relaxed selection in *P. azurophilum* and *P. leucocytica* as well. This is an unexpected result as we hypothesize that these digestive enzymes have enzymatic roles outside of hemoglobin and therefore would not expect to see relaxation in selection pressures.

Relaxed selection was also detected in these genes in *P. floridense* relative to the bird and mammal malaria parasites. As hemoglobin digestion and hemozoin formation clearly do occur in *P. floridense* , these genes were originally tested as a control comparison and no relaxed selection was expected. The data imply that there are relative differences in selection along these genes in all three lizard malaria parasites regardless of hemoglobin digestion. More genetic data from other lizard‐infective *Plasmodium* species within the sauropsid clade would help elucidate whether this pattern of relaxed selection for these genes is common across this clade or specific to this Caribbean lizard infection system.

Three genes (EPS15, dipeptidyl aminopeptidase 1, plasmepsin IV) show different signatures of selection in *P. azurophilum* and *P. floridense* (RBC‐infecting) versus *P. leucocytica* (WBC‐infecting) relative to the entire *Plasmodium* tree. This implies that the cellular environment might be a stronger driver of selection pressure on these genes rather than the lack of hemoglobin digestion. Because these three genes show different selective pressures in *P. leucocytica*, which infects white blood cells, compared to the two red blood cell‐inhabiting species, we hypothesize that these three genes may play different roles in each cellular environment.

Some of the transcribed gene sequences from the lizard malaria transcriptomes did not span the length of the entire coding sequence. As a result, the entire alignment had to be trimmed for all the sequences (see Section 4). Further sequencing should be completed for these parasite genes and these selection tests should be re‐run using the entire transcript sequence for an even more robust confirmation of these conclusions. Given the data we currently have, however, a decrease in selective pressure is hypothesized and these proteins may not be as functionally important for these parasites in the future. These parasites could also have evolved alternative (currently unknown) enzymes with similar functions. Additional proteins with redundant function could lead to the reduction in selective pressure on these genes.

These genes are not yet pseudogenes, however, especially as seen in *P. floridense* , which digests hemoglobin and produces hemozoin. Additionally, as lizards have nucleated red blood cells, the cytosol of a lizard red blood cell could be composed of proteins other than hemoglobin that these enzymes could be acting on. Indeed, lizard red blood cells may vary greatly from mammalian red blood cells, as their life span alone is drastically longer (600–800 days compared to 120 days) (Claver and Quaglia 2009). Future genetic manipulations/protein functional analyses would help determine the essentiality of these proteins.

### 3.3 Is There a Selective Advantage to Not Digest Hemoglobin?

Given that *P. leucocytica* inhabits a white blood cell and has no hemoglobin to digest, lack of hemozoin is not unexpected. *P. azurophilum*, however, still has access to hemoglobin and the capability to digest it as it retains and transcribes the necessary genes. The lack of hemozoin in *P. azurophilum* indicates that the parasite either does not digest hemoglobin or it digests hemoglobin without producing hemozoin (Figure 1C). In the latter potential case, there must be some other method by which the parasite deals with the buildup of toxic heme. It is possible that the parasite takes advantage of the lizard host heme oxygenase, however, functional analyses on the lizard enzyme would be necessary. Analysis of parasite transporter proteins would also be interesting as that would provide insight to whether the parasite is importing the lizard heme oxygenase into its cell or exporting the heme from its cell to the host environment for detoxification.

The role of hemozoin in the host's immune response has been debated, but there is new evidence that parasite DNA bound to hemozoin accumulates in the liver and recruits nonresident macrophages. This immune response was also shown to provide protection against re‐infection for a short period, leading the authors to hypothesize that this may play a role in individuals living in malaria endemic regions who confer concomitant immunity (Franco et al. 2023). The *Plasmodium*‐infected lizards in the Caribbean appear to live with chronic infections and often have multi‐infections (Table S1). We hypothesize that a reduction of hemozoin production (and, therefore, host immune response) is beneficial for co‐infections to establish and persist. Once this trait evolved, this could have allowed for the ancestral parasite to persist in white blood cells and speciate into extant *P. azurophilum* and *P. leucocytica*.

### 3.4 A Case for Understudied Parasites: Comparative Selection Analyses Using Non‐Mammalian Genetic Data Are Still Beneficial for Human Malarial Studies

Most of *Plasmodium* (genetic) research is completed in mammalian parasites (especially in *P. falciparum*). As a result, the data for the lizard malaria parasites in this study are being compared to known data from their mammalian counterparts. The ancestral host for *Plasmodium* species, however, likely was a bird or lizard (Galen et al. 2018), both of which have nucleated red blood cells. The ancestral hemoglobin digestion pathway may actually have been more similar to these sauropsid parasites rather than the mammalian parasites. A more intuitive comparison, therefore, may be to look at what has changed in the mammalian *Plasmodium* clade relative to the sauropsid clade. The results from the relaxed selection tests support this as seven genes show signatures of intensification/increased selective pressures in the mammalian parasites relative to the rest of the tree (Table 3).

Given the role this pathway plays in antimalarial drug activation and antimalarial resistance, comparative selection analyses could help identify future drug targets. Proteins that are conserved across *Plasmodium*, essential in mammalian parasites (Table 2), and not undergoing shifts in selection (Table 3) could be potential drug targets. Genes undergoing relaxed selection may no longer be necessary for the parasite and would not be a good drug target. Conversely, genes undergoing intensification of selective pressures may be in the process of evolving and could result in drug resistance in the future. For example, Ap2μ shows signatures of selection intensification in the mammalian clade (Table 3). These tests were conducted using sequences from the *P. falciparum* (Pf3D7) reference genome, which was published in 2002 (Gardner et al. 2002). Mutations in Ap2μ that confer ART resistance were reported in *P. falciparum* parasites in 2014 and 2019 (Henriques et al. 2014; Henrici et al. 2019), indicating that selection intensification likely preceded the rise of resistant mutations. In addition to analyzing protein space (Ogbunugafor 2020), selection tests could equally help inform and act as harbingers of resistance evolution. This test is not possible without comparison data, however. More data across the *Plasmodium* phylogenetic tree, but especially in the sauropsid clade, is essential to continue making these comparisons between mammalian and sauropsid *Plasmodium*.

## 4 Materials and Methods

### 4.1 Sample Collection

Fieldwork was completed in September 2018 on Saba Island in the northeastern Caribbean. All fieldwork conducted with lizards was approved by the American Museum of Natural History Institutional Animal Care and Use Committee (AMNH_Perkins_2014). Sampling permits were approved by the Saba Conservation Foundation and The Executive Council of the Public Entity Saba (Permit No. 488/2018). Adult *A. sabanus* lizards (male & female) were captured using a slip lasso. Blood was collected via toe clippings for blood smears on slides and blood dots on filter paper for DNA extraction. Slides were fixed in methanol and stained with Hema3. Infection status was determined in the field for each lizard by scanning approximately 1000 blood cells at 1000×. Blood samples from toe clippings were also collected from some lizards depending on infection status and stored in the Qiagen RNeasy Protect Animal Blood Tubes for RNA extraction. Samples were stored at −20°C in the field, and RNA samples were moved to −80°C in the lab. All lizards were returned to their original collection site within 24 h.

### 4.2 Sample Processing and Sequencing

DNA was extracted from blood dots on filter paper using the Qiagen DNeasy Blood & Tissue Kit. Infection status (initially assessed by microscopy) was confirmed for each sample via polymerase chain reaction (PCR) using primers for *Plasmodium* mitochondrial genes (Figure S7, Table S4). PCR products were Sanger sequenced on an Applied Biosystems 3730xl DNA analyzer for confirmation and multi‐infection detection (indicated by double peaks on chromatogram).

RNA was isolated from the blood samples stored in the Qiagen RNeasy Protect Animal Blood Tubes with the Thermo Fisher PicoPure RNA Isolation kit. RNA concentration and quality were assessed with a Thermo Fisher Qubit and an Agilent BioAnalyzer. The Takara Bio SMART‐Seq v.4 Ultra Low Input RNA for Sequencing kit was used for cDNA synthesis on the acceptable RNA samples. cDNA quality was also assessed with a BioAnalyzer. Fifteen cDNA samples were sent to Genewiz (South Plainfield, NJ) for library prep (Illumina Nextera XT DNA Library Preparation Kit) and sequencing on an Illumina HiSeq 4000 (2 × 150 bp, 1 lane).

### 4.3 Parasite Transcriptome Assembly and Analysis

Raw reads were quality assessed with FastQC v.0.11.8 (Andrews 2010). Reads were trimmed and adapter sequences were removed with TrimGalore v.0.6.4 (Krueger 2016). Meta‐transcriptomes were assembled de novo in Trinity v.2.8.5 (Grabherr et al. 2011) for each of the three *Plasmodium* species. The meta‐assemblies were filtered using ContamFinder (Borner and Burmester 2017) to select for parasite contigs only and, therefore, remove any contaminating host contigs. Assemblies were annotated for open reading frames using TransDecoder v.5.5.0 (Haas 2012). Expression counts (transcripts per million, TPM) were calculated for every sample by mapping the sample reads back to the three meta‐assemblies with RSEM v.1.3.1 (Li and Dewey 2011) and Bowtie2 v.2.3.5 (Langmead and Salzberg 2012). Samples were mapped to each meta‐assembly individually to account for lizards with multi‐infections. RSEM output (TPM values) were imported into R and analyzed with the DESeq2 package (Love et al. 2014). RSEM output was filtered using a cutoff value of TPM > 1 and normalized using the variance stabilizing transformation (vst) function within the DESeq2 package. Plots (PCA, screeplot, heatmap) were constructed in DESeq2, Pheatmap, and PCAtools (Love et al. 2014; Kolde 2019; Blighe and Lun 2022).

### 4.4 Life Stage Gene Expression

To better characterize which parasite life stages make up each field sample, a statistical approach called gene expression deconvolution was performed. This is a mathematical model to estimate cell composition in bulk RNAseq samples using gene expression profiles from reference datasets. Deconvolution of samples was conducted in CIBERSORTx (Newman et al. 2019) using a published *Plasmodium* reference data set (Tebben et al. 2022). As described above, RSEM output (TPM values) for the bulk RNAseq samples was imported into R and analyzed with the DESeq2 package (Love et al. 2014). DESeq2 was used to construct a count matrix with gene expression counts for each sample (after filtering with a cutoff value of TPM > 1). Using the orthologous codes in PlasmoDB (The Plasmodium Genome Database Collaborative 2001), the corresponding PF3D7 gene IDs were merged with our gene expression count matrix so that the published reference data set (Tebben et al. 2022) could be utilized. The gene expression matrix was then imported into CIBERSORTx (Newman et al. 2019) and run under “impute cell fractions” with a custom analysis and the S‐mode batch correction using the published reference files (Tebben et al. 2022). A stacked bar chart depicting estimated relative abundances of parasite life stages in each sample was constructed in the CIBERSORTx output.

### 4.5 Hemoglobin Digestion Gene Expression & Selection Tests

A heatmap for expression of hemoglobin digestion genes was constructed in DESeq2 (Love et al. 2014) and Pheatmap (Kolde 2019) after data filtering (described previously). Orthologous coding sequences from the laverania, vinckei, and sauropsid *Plasmodium* clades (Galen et al. 2018) were obtained from PlasmoDB (https://www.plasmodb.org) (The Plasmodium Genome Database Collaborative 2001) for each gene alignment. *Plasmodium* species from the macaque clade (i.e., *Plasmodium vivax*) were excluded from the final analysis as the disproportionately high GC content in these species relative to the other clades was found to skew the alignments. *Plasmodium* parasites are uniquely AT rich (even relative to other haemosporidians) (Galen et al. 2018). This trait is found throughout the *Plasmodium* genus apart from the macaque clade, which is trending towards becoming a significantly GC rich genome (Nikbakht 2014).

Multiple sequence alignments were constructed using a codon aware pipeline (Pond 2019) with MUSCLE (Edgar 2004). Alignments were trimmed by hand in Geneious Prime v.2019.0.4. For comparison, alignments were also trimmed using the automated function in trimAL (Capella‐Gutierrez et al. 2009), however, these alignments resulted in slightly lower confidence maximum likelihood trees and were not used further. Due to missing data in the transcriptomes, some gene sequences from the lizard‐infective parasite transcriptomes do not span the entire predicted coding sequence. Even if only one species' gene sequence was cut off, the entire alignment was trimmed to the length of the shorter sequence to avoid skewed results in downstream analyses. The length at which the alignments were trimmed was determined by maximizing keeping as much of the sequence as possible and having high bootstrap values on the phylogenetic tree. These alignments include Kelch13, Ap2μ, EPS15, DPAP1, falcilysin, and M1‐family alanyl aminopeptidase. Maximum likelihood (ML) trees were constructed with the edited alignments in RAxML (Stamatakis 2014). Modeltest was run in RAxML to determine the best fit model for tree construction for each alignment. 1000 replicates were completed for each tree. Relaxed selection was tested along the test branches of the phylogenetic trees (relative to reference branches) using RELAX (Wertheim et al. 2015) in HyPhy v.2.5.48 (via the command line) (Weaver et al. 2018). HyPhy was conducted with the “Ciliate‐Nuclear” genetic code as malaria codon usage differs from mammalian organisms (as updated by Wang et al. (2022)). Several evolutionary forces including the high AT richness in malaria genomes have driven the parasites to a biased codon usage (Sinha and Woodrow 2018).

In this study, we tested for selection intensity across multiple genes within one well‐documented pathway. This limits the randomness associated with statistical significance to some degree, which thereby likely limits the false positive discovery rate. Therefore, we have reported raw *p* values extracted from the RELAX test (Table 3). A multiple comparisons correction is more frequently required for genome wide exploratory assessments of selection intensity where no hypothesis‐driven tests are predesignated, as argued by Anisimova and Yang (Anisimova and Yang 2007) for example. Further, in this study, the outcome of the RELAX test is interpreted for each gene individually, rather than altogether as a collective pathway. Conceptually, this is because while each individual gene has the capacity to be involved in alternative functions, they are not likely to all be co‐dependent on each other. The extent of the biological roles of each of these genes is thus (potentially) not dependent on one another from an evolutionary perspective, and the holistic overview of the data observed here indeed suggests that is the case. In situations like this, Rubin (Rubin 2021) argues that multiple comparison corrections are not necessary and in fact can be deleterious to the data representation.

### 4.6 Orthologous Gene Searches in Other Apicomplexan Parasites

Potential orthologous proteins for hemoglobin digestion genes in related apicomplexan parasites, *Babesia*, *Theileria*, and *Toxoplasma* species, were determined via a protein blast search. Protein translations for each gene from the *P. falciparum* 3D7 genome were obtained from PlasmoDB (The Plasmodium Genome Database Collaborative 2001) and utilized as the search queries. These translated protein sequences were searched against the publicly available *Babesia bigemina* reference proteome (UniProt ID UP000033188), *Theileria parv*a reference proteome (UniProt ID UP000001949), and *Toxoplasma gondii* reference proteome (UniProt ID UP000557509) (UniProt Consortium 2023). Blast searches were conducted in DIAMOND v.2.1.9 (Buchfink et al. 2021) due to its efficiency (Hernandez‐Salmeron and Moreno‐Hagelsieb 2020).

### 4.7 Protein Structure Prediction

Protein sequences were obtained after the amino acid translation step in the codon aware alignment pipeline (using the “Ciliate‐Nuclear” code) (Pond 2019). Structures for heme detoxification protein for *P. floridense* , *P. azurophilum*, and *P. leucocytica* were predicted using AlphaFold2 (Jumper et al. 2021) via ColabFold v.1.5.2 (Mirdita et al. 2022). For each protein sequence, a multiple sequence alignment was constructed in ColabFold via MMseq2 using the uniref and environmental sequence database (Suzek et al. 2007). Five models were run using the alphafold_ptm2 model type for monomer prediction. The model was recycled six times for improvement and the best ranked model (by pLDDT) was refined using AMBER (Hornak et al. 2006). The best ranked model was then imported into ChimeraX v.1.6.1 (Goddard et al. 2018), and the root mean square deviation (RMSD) in the Cα positions was calculated for the model relative to the predicted protein structure for *P. falciparum* 3D7 heme detoxification protein (obtained from AlphaFold; Jumper et al. 2021).

## Author Contributions

**Sarah J. Pangburn:** conceptualization (equal), data curation (equal), formal analysis (lead), investigation (lead), methodology (equal), writing – original draft (lead), writing – review and editing (lead). **Janus Borner:** conceptualization (equal), data curation (lead), formal analysis (equal), investigation (equal), methodology (equal), writing – review and editing (supporting). **Sidhanth Misra:** formal analysis (supporting). **Jessica Zavalunova:** formal analysis (supporting). **Susan L. Perkins:** conceptualization (lead), funding acquisition (lead), investigation (equal), methodology (equal), writing – review and editing (supporting).

## Funding

This work was supported by the American Museum of Natural History (1R03AI117223‐01A1, 5R03AI117223‐02), Bundesministerium für Forschung, Technologie und Raumfahrt (BMFTR), and the European Union.

## Conflicts of Interest

The authors declare no conflicts of interest.

## Supporting information


**Figure S1:** Parasitemia microscopy counts for 2018 field samples. The number of *Plasmodium azurophilum* and *Plasmodium floridense* infected red blood cells (RBCs) were counted out of 10,000 RBC. The number of *Plasmodium leucocytica* infected azurophils (white blood cells, WBCs) was calculated for the number of azurophils seen within the same fields of view as the 10,000 RBC count. There are far fewer WBCs seen in the blood smears relative to RBCs, which is why the percentage of infected cells appears much higher in *P. leucocytica*. If the counts were to be shown as the number of infected WBCs/10,000 RBCs, the parasitemia levels would be equivalent to the other species. Only samples with parasites above a detectable load by eye are represented here. Graph was constructed using GraphPad Prism v.9.4.1.
**Figure S2:** Principal component analysis of *Plasmodium* species transcriptome samples labeled by infection status. Bulk RNAseq was performed on *Anolis sabanus* blood infected with *Plasmodium floridense*, *Plasmodium azurophilum*, and *Plasmodium leucocytica*. Parasite meta‐transcriptomes for all three species were constructed, and transcript read counts were calculated using these meta‐assemblies for twelve lizard samples. Some of the samples were infected with only one parasite species (dark purple) while the others were infected with multiple species of parasites (cornflower blue). Samples (top 500 genes) still cluster by parasite species rather than whether or not they were a single infection or part of a multi‐infection. Infection status (single vs. multi) may play a role for within‐species variation. The first two principal components account for 89% of variation. Principal component analysis was performed using the DESeq2 and ggplot2 packages in R (Love et al. 2014; Wickham 2016). Plot colors were constructed using “Archambault” from the MetBrewer R package (Mills 2022).
**Figure S3:** Principal component analysis scree plot. Principal component analysis was performed on *Plasmodium* transcriptome samples using the PCAtools R package (Blighe and Lun 2022). The first two principal components explain most of the variation across all samples.
**Figure S4:** Principal component analysis with top 5 differing transcripts labeled. Principal component analysis was performed on the parasite transcriptomes using the PCAtools R package (Blighe and Lun 2022). The top 5 transcripts driving the variation for each principal component are labeled on the plot. The transcripts are labeled using the gene ID for the *Plasmodium gallinaceum* ortholog, which was obtained from PlasmoDB (The Plasmodium Genome Database Collaborative 2001). The first two principal components account for 68.46% of variation.
**Figure S5:** Maximum likelihood tree for falcipain and plasmepsin genes. (A) Potential falcipain orthologs from *Plasmodium floridense*, *Plasmodium azurophilum*, and *Plasmodium leucocytica* were aligned with sequences for *Plasmodium falciparum* falcipain 1, 2a, 2b, and 3. Falcipain 1 and cysteine protease sequences from *Plasmodium gallinaceum* and *Plasmodium relictum* were also included. *Haemoproteus tartakovskyi* is used as an outgroup. (B) Putative plasmepsin orthologs from *Plasmodium floridense*, *Plasmodium azurophilum*, and *Plasmodium leucocytica* were aligned with sequences for *Plasmodium falciparum* plasmepsin 1–4 and 6. Sequences for *Plasmodium gallinaceum* and *Plasmodium relictum* plasmepsin 4 and 6 were also included. Orthologous sequences were obtained from PlasmoDB (The Plasmodium Genome Database Collaborative 2001). Maximum likelihood tree constructed in RAxML (Stamatakis 2014). Support (bootstrap values) is indicated along the nodes, and the branch length is indicated by the key at the bottom.
**Figure S6:** Maximum likelihood trees for genes involved in hemoglobin digestion. Maximum likelihood trees were constructed in RAxML (Stamatakis 2014) for each gene involved in the hemoglobin uptake and digestion pathway. Support (bootstrap values) is indicated along the nodes, and the branch length is indicated by the key at the bottom. Orthologous sequences (Table S3) were obtained from PlasmoDB (The Plasmodium Genome Database Collaborative 2001). *Haemoproteus tartakovskyi* is used as an outgroup.
**Figure S7:** PCR primers for diagnosing infection status & their positions along the *Plasmodium* mitochondrial genome. Mitochondrial genomes for all three parasite species were sequenced using a variety of primers (Table S4) and aligned to the *Plasmodium relictum* SGS1 mitochondrial genome (GenBank: LN835311.1) in Geneious Prime (Version 2019.0.4). A schematic of the mitochondrial genome is displayed with the cytochrome oxidase subunit I gene and cytochrome B gene positioning labeled. PCR primers used for diagnosing sample infection status target the COI and CytB genes. Forward primers are labeled in forest green and reverse primers are labeled in lime green.
**Table S1:** Transcriptome sample information. The lizard sample number, sex of the lizard, the snout ventricle length measurement (SVL, mm, proxy for lizard age), and *Plasmodium *infection status (diagnosed via microscopy and PCR vs. transcriptomic sequencing) is listed for each sample.
**Table S2:** Potential orthologous proteins for known hemoglobin digestion genes in other apicomplexan parasites. Orthologous protein sequences were obtained by blasting the *Babesia bigemina*, *Theileria parva*, and *Toxoplasma gondii* proteomes with *Plasmodium falciparum* 3D7 protein sequences for each gene involved in hemoglobin digestion. The protein ID for each blast hit is listed as well as the associating *e*‐value and bit score.
**Table S3:** Orthologous sequence codes. Orthologous sequences were obtained from PlasmoDB (The Plasmodium Genome Database Collaborative 2001) for each gene analyzed (phylogenetic and selection analysis) in the hemoglobin uptake and digestion pathway. Representative species were chosen for the laverania (apes), vinckei (rodents), and sauropsid (birds, lizards) clades (Galen et al. 2018) in the *Plasmodium* phylogenetic tree.
**Table S4:** Polymerase chain reaction (PCR) primers used to sequence the mitochondrial genomes and diagnose infection status. PCRs and sanger sequencing were completed using multiple primer sets to confirm infection status of each field sample. Primer sequences were obtained from S. L. Perkins (unpublished), Hellgren et al. (2004), Creasey et al. (1993), Perkins and Schall (2002), Perkins and Austin (2009), Pacheco et al. (2018), Perkins (2008), or designed in this study.

## Data Availability

The data that support the findings of this study are openly available in NCBI Sequence Read Archive at https://www.ncbi.nlm.nih.gov/sra, reference number PRJNA1356547.
