# Supplementary material for: Hemoglobin Digestion Genes Are Conserved in Lizard‐Infective Plasmodium Species With Different Host Cellular Niches
Source: Ecol Evol. 2026 Jun 8;16(6):e73801. doi: 10.1002/ece3.73801 (PMC13244081; doi:10.1002/ece3.73801)
Supplement: Supplementary file 1 — Figure S1: Parasitemia microscopy counts for 2018 field samples. The number of Plasmodium azurophilum and Plasmodium floridense infected red blood cells (RBCs) were counted out of 10,000 RBC. The number of Plasmodium leucocytica infected azurophils (white blood cells, WBCs) was calculated for the number of azurophils seen within the same fields of view as the 10,000 RBC count. There are far fewer WBCs seen in the blood smears relative to RBCs, which is why the percentage of infected cells appears much higher in P. leucocytica. If the counts were to be shown as the number of infected WBCs/10,000 RBCs, the parasitemia levels would be equivalent to the other species. Only samples with parasites above a detectable load by eye are represented here. Graph was constructed using GraphPad Prism v.9.4.1. Figure S2: Principal component analysis of Plasmodium species transcriptome samples labeled by infection status. Bulk RNAseq was performed on Anolis sabanus blood infected with Plasmodium floridense, Plasmodium azurophilum, and Plasmodium leucocytica. Parasite meta‐transcriptomes for all three species were constructed, and transcript read counts were calculated using these meta‐assemblies for twelve lizard samples. Some of the samples were infected with only one parasite species (dark purple) while the others were infected with multiple species of parasites (cornflower blue). Samples (top 500 genes) still cluster by parasite species rather than whether or not they were a single infection or part of a multi‐infection. Infection status (single vs. multi) may play a role for within‐species variation. The first two principal components account for 89% of variation. Principal component analysis was performed using the DESeq2 and ggplot2 packages in R (Love et al. 2014; Wickham 2016). Plot colors were constructed using “Archambault” from the MetBrewer R package (Mills 2022). Figure S3: Principal component analysis scree plot. Principal component analysis was performed on Plasmodium [file ECE3-16-e73801-s001.pdf]

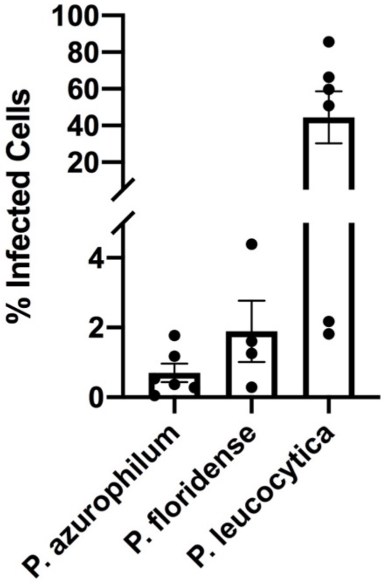
100
80
60
40
20
4
2
0
% Infected Cells
P. azurophilum
P. floridense
P. leucocytica

S1 Fig

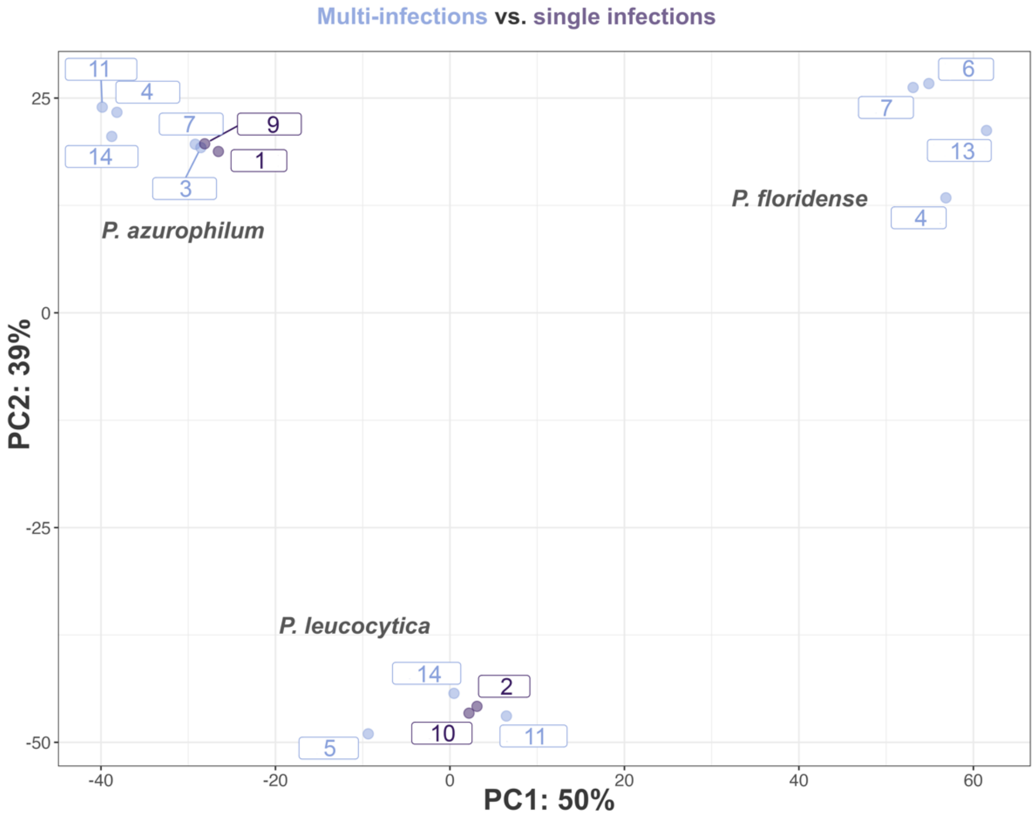
Multi-infections vs. single infections
PC2: 39%
PC1: 50%
P. azurophilum
P. floridense
P. leucocytica
11
4
7
9
14
1
3
6
7
13
4
14
2
10
5
11
25
0
-25
-50
-40
-20
0
20
40
60

S2 Fig

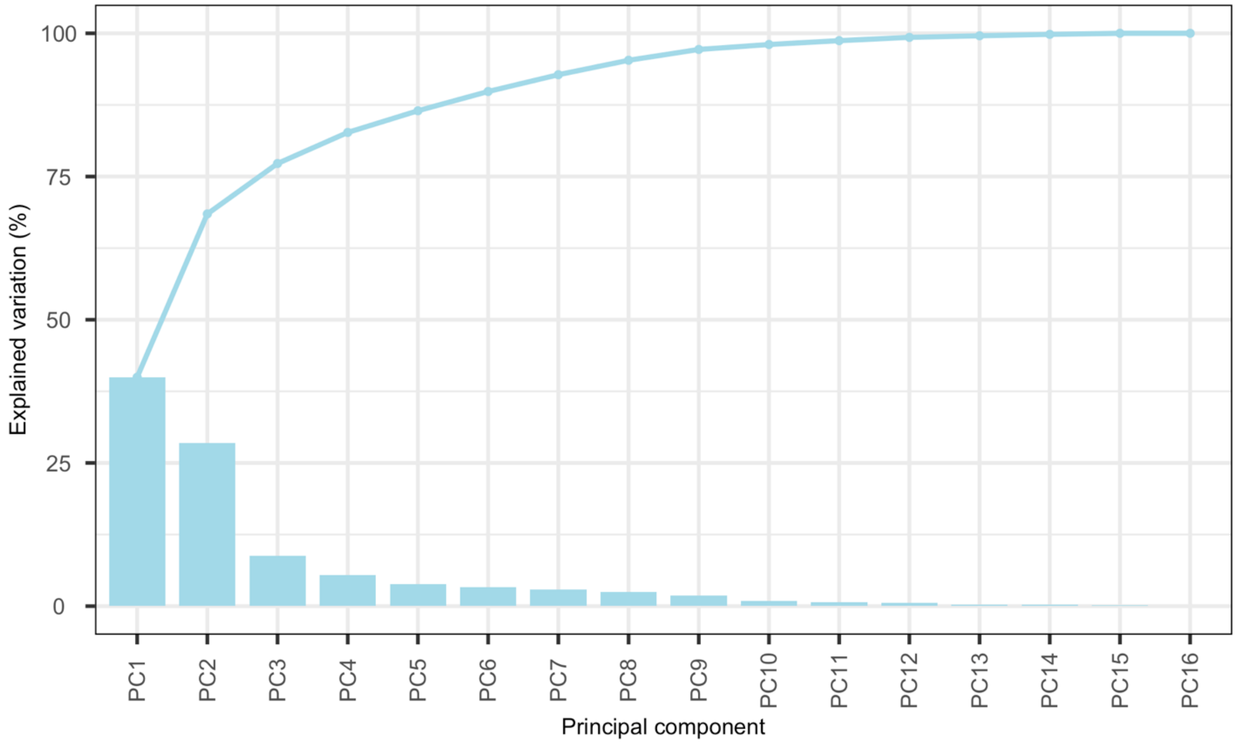
100
75
50
25
0
Explained variation (%)
PC1
PC2
PC3
PC4
PC5
PC6
PC7
PC8
PC9
PC10
PC11
PC12
PC13
PC14
PC15
PC16
Principal component

S3 Fig

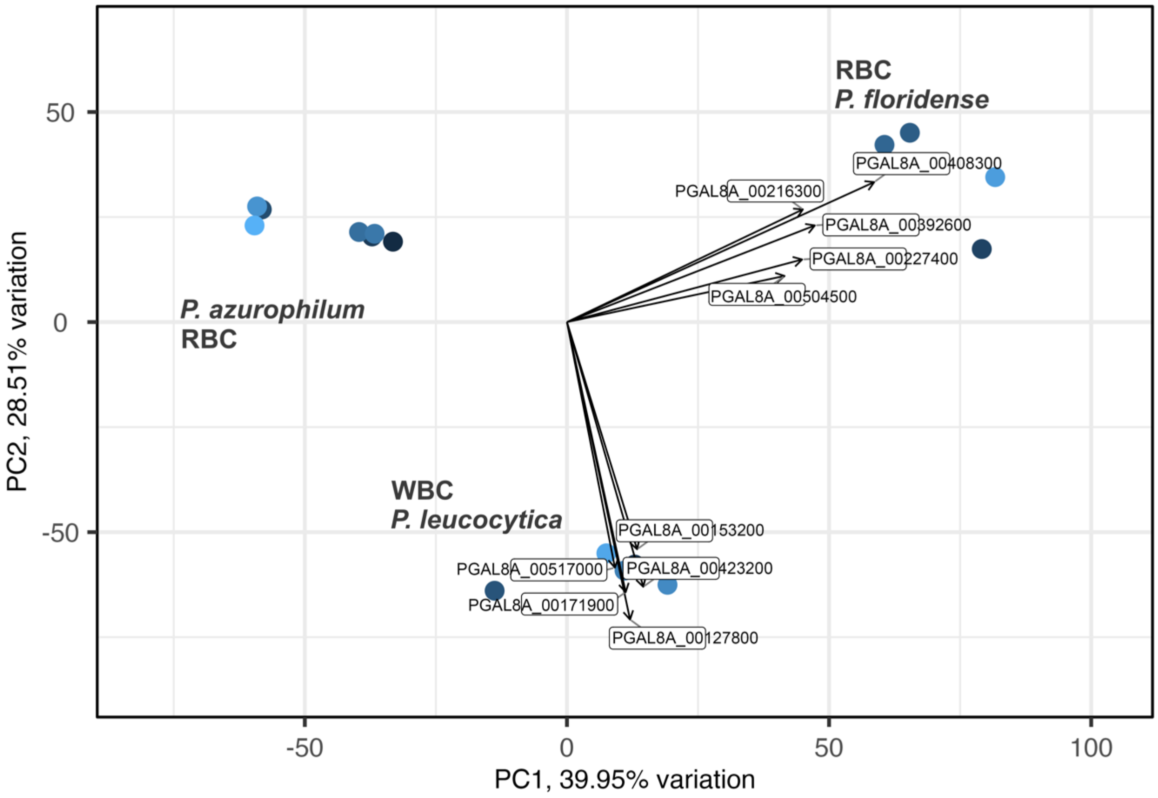
RBC
P. floridense
P. azurophilum
RBC
WBC
P. leucocytica
PGAL8A_00408300
PGAL8A_00216300
PGAL8A_00392600
PGAL8A_00227400
PGAL8A_00504500
PGAL8A_00153200
PGAL8A_00517000
PGAL8A_00423200
PGAL8A_00171900
PGAL8A_00127800
PC2, 28.51% variation
PC1, 39.95% variation
50
0
-50
-50
0
50
100

S4 Fig

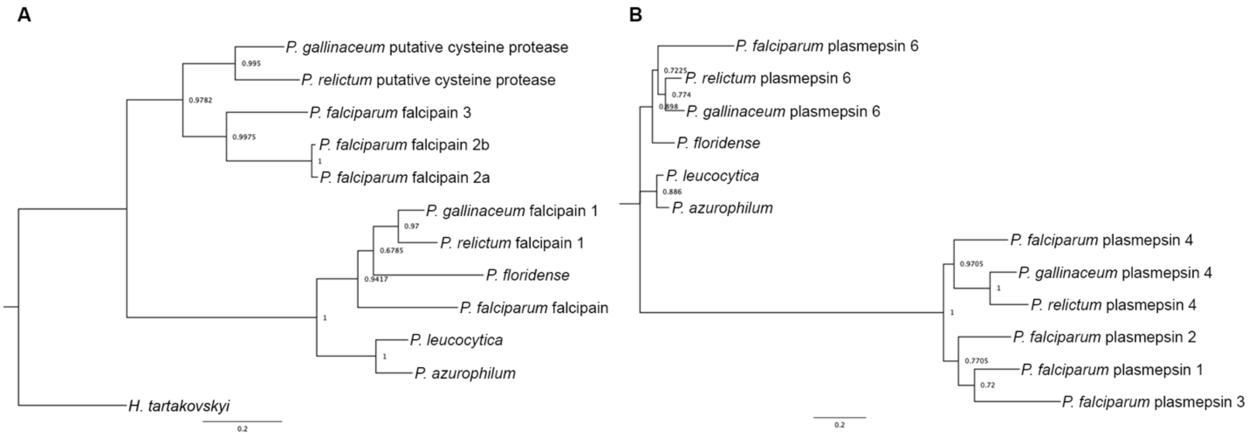
A
P. gallinaceum putative cysteine protease
P. relictum putative cysteine protease
P. falciparum falcipain 3
P. falciparum falcipain 2b
P. falciparum falcipain 2a
P. gallinaceum falcipain 1
P. relictum falcipain 1
P. floridense
P. falciparum falcipain
P. leucocytica
P. azurophilum
H. tartakovskyi
0.995
0.9782
0.9975
0.97
0.6785
0.9417
0.2
B
P. falciparum plasmepsin 6
P. relictum plasmepsin 6
P. gallinaceum plasmepsin 6
P. floridense
P. leucocytica
P. azurophilum
P. falciparum plasmepsin 4
P. gallinaceum plasmepsin 4
P. relictum plasmepsin 4
P. falciparum plasmepsin 2
P. falciparum plasmepsin 1
P. falciparum plasmepsin 3
0.7225
0.774
0.886
0.9705
0.7705
0.72
0.2

S5 Fig

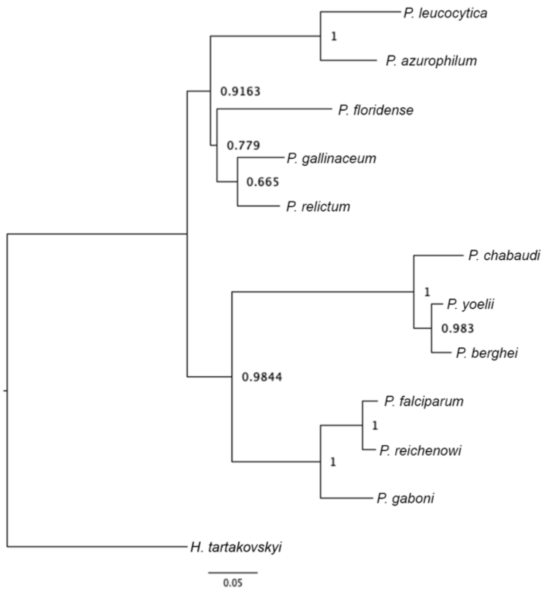

Ap2 Complex Subunit μ (Ap2μ)

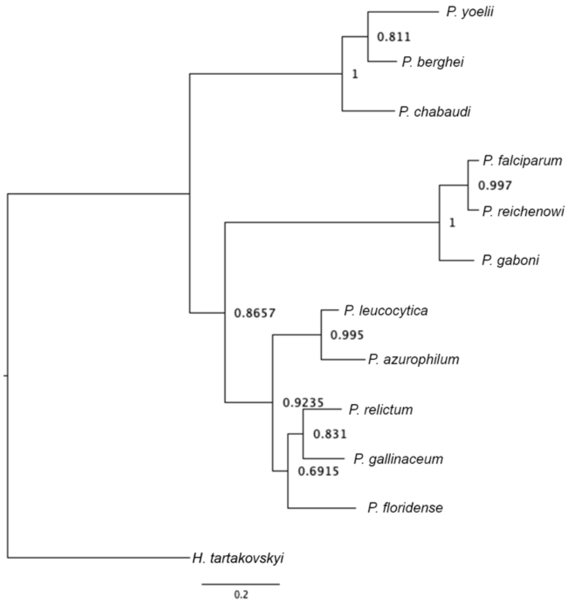

EPS15-like Protein (EPS15)

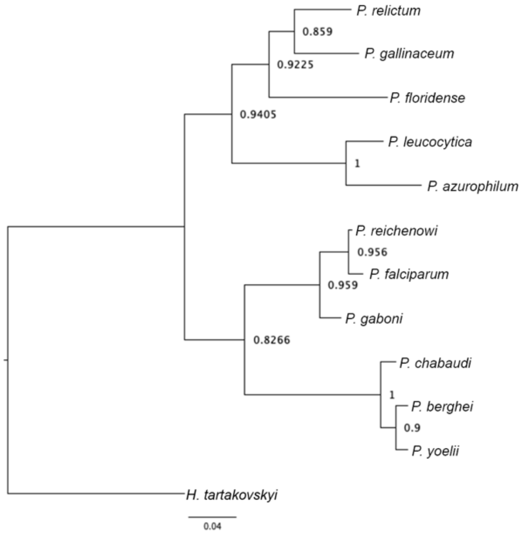

Kelch13 (K13)

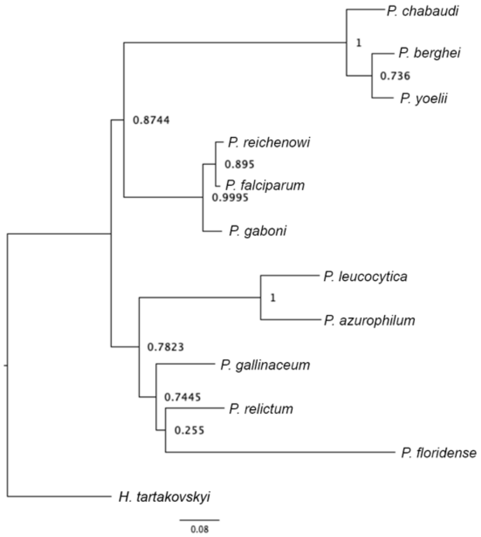

Plasmepsin IV (PM4)

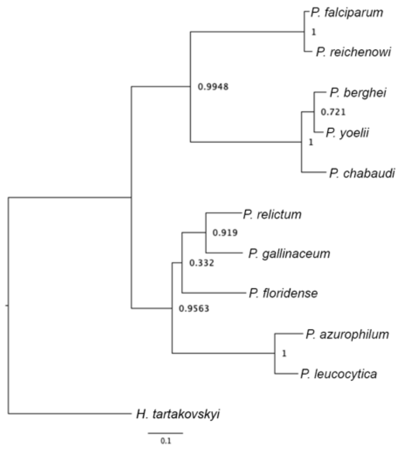

Dipeptidyl Aminopeptidase 1 (DPAP1)

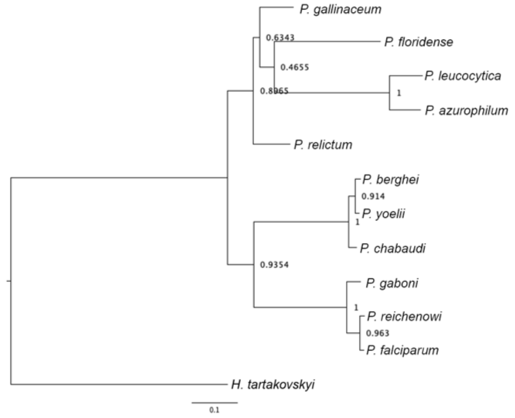

Falcilysin (FLN)

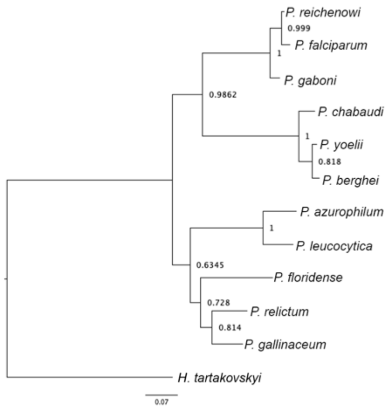

M1-family alanyl aminopeptidase (M1AAP)

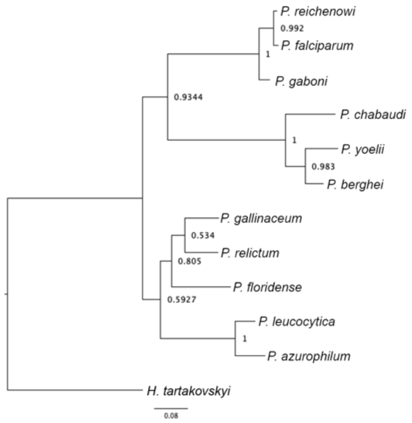

M17 leucyl aminopeptidase (LAP)

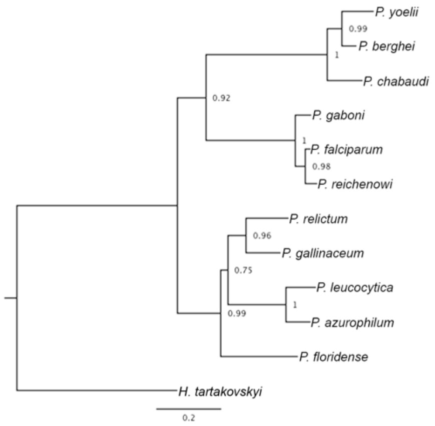

Aminopeptidase P (APP)

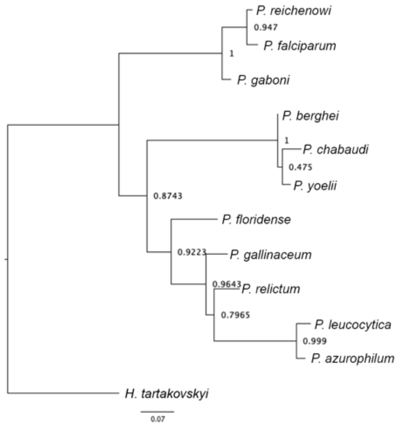

Heme Detoxification Protein (HDP)

S6 Fig

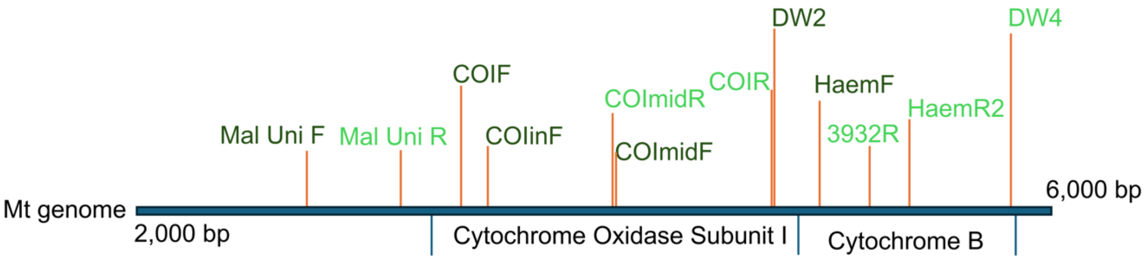
DW2
DW4
COIF
COIR
HaemF
COImidR
HaemR2
Mal Uni F
Mal Uni R
COIinF
3932R
COImidF
6,000 bp
Mt genome
2,000 bp
Cytochrome Oxidase Subunit I
Cytochrome B

S7 Fig

S1 Table

**Sample**	**Sex**	**SVL (mm)**	**Infection Diagnosis (microscopy + PCR)**	**Infection Diagnosis (transcriptomic data)**
**1**	M	55	*P. azurophilum*	*P. azurophilum*
**2**	M	53	*P. leucocytica*	*P. leucocytica*
**3**	M	48	*P. azurophilum*	*P. azurophilum*; *P. leucocytica*
**4**	M	60	*P. floridense*; *P. azurophilum*	*P. floridense*; *P. azurophilum; P. leucocytica*
**5**	M	56	*P. floridense*; *P. leucocytica*	*P. floridense*; *P. leucocytica; P. azurophilum*
**6**	M	61	*P. floridense*	*P. floridense*; *P. leucocytica*
**7**	M	64	*P. floridense*	*P. floridense; P. azurophilum*
**8**	F	44	uninfected	*P. leucocytica*
**9**	M	50	*P. azurophilum*	*P. azurophilum*
**10**	M	52	*P. leucocytica*	*P. leucocytica*
**11**	M	59	*P. leucocytica; P. azurophilum*	*P. leucocytica; P. azurophilum*
**12**	M	60	uninfected	*P. leucocytica; P. floridense*
**13**	F	50	*P. floridense*; *P. leucocytica*	*P. floridense*; *P. leucocytica; P. azurophilum*
**14**	F	46	*P. leucocytica*	*P. leucocytica; P. azurophilum*
**15**	F	47	uninfected	uninfected

S2 Table

P. falciparum Gene Query	Target Species Proteome	Protein ID	*e*-value	Bit Score
**Kelch13 (K13)**	*Babesia bigemina*	A0A061DCK8_BABBI	3.60E-87	317.8
**Ubiquitin Carboxyl-terminal Hydrolase 1 (UBP1)**	*Babesia bigemina*	A0A061D6S5_BABBI	2.10E-47	188
**Cysteine Proteinase Falcipain 2a (FP2A)**	*Babesia bigemina*	C0A1J0_BABBI	4.00E-42	167.5
	Babesia bigemina	A0A061D1B9_BABBI	4.40E-41	164.1
	Babesia bigemina	A0A061CZ03_BABBI	2.70E-38	154.8
**Cysteine Proteinase Falcipain 2b (FP2B)**	*Babesia bigemina*	C0A1J0_BABBI	1.20E-41	166
	Babesia bigemina	A0A061D1B9_BABBI	4.90E-40	160.6
	Babesia bigemina	A0A061CZ03_BABBI	9.20E-39	156.4
**Plasmepsin II (PMII)**	*Babesia bigemina*	A0A061D2V4_BABBI	7.90E-08	53.5
**Plasmepsin IV (PM4)**	*Babesia bigemina*	A0A061DEM2_BABBI	4.10E-33	137.5
	Babesia bigemina	A0A061DBB1_BABBI	2.50E-22	101.7
**Heme Detoxification Protein (HDP)**	*Babesia bigemina*	A0A061DBU8_BABBI	6.00E-40	159.1
**Falcilysin (FLN)**	*Babesia bigemina*	A0A061D8Z5_BABBI	1.30E-113	406.4
**M1-family alanyl aminopeptidase (M1AAP)**	*Babesia bigemina*	A0A061DCR4_BABBI	2.10E-155	545
**M17 leucyl aminopeptidase (LAP)**	*Babesia bigemina*	A0A061D439_BABBI	8.40E-98	352.8
**Aminopeptidase P (APP)**	*Babesia bigemina*	A0A061DDX0_BABBI	2.30E-92	335.1
**Dipeptidyl Aminopeptidase 1 (DPAP1)**	*Babesia bigemina*	A0A061DB97_BABBI	4.60E-31	131.3
	Babesia bigemina	A0A061DD89_BABBI	1.40E-16	83.2
**Kelch13 (K13)**	*Theileria parva*	Q4N8I3_THEPA	8.90E-94	339
	Theileria parva	Q4N3L4_THEPA	5.30E-06	47.4

P. falciparum Gene Query	Target Species Proteome	Protein ID	*e*-value	Bit Score
**Ubiquitin Carboxyl-terminal Hydrolase 1 (UBP1)**	*Theileria parva*	Q4MZ16_THEPA	1.10E-53	208
**KIC7 (KIC7)**	*Theileria parva*	Q4N024_THEPA	1.60E-11	65.5
**Cysteine Proteinase Falcipain 2a (FP2A)**	*Theileria parva*	Q4N068_THEPA	3.80E-48	186.8
	Theileria parva	Q4N640_THEPA	2.60E-41	164.1
	Theileria parva	Q4N069_THEPA	5.10E-37	149.8
	Theileria parva	Q4N067_THEPA	1.30E-35	145.2
	Theileria parva	Q4N639_THEPA	5.30E-34	139.8
	Theileria parva	Q4N638_THEPA	2.20E-32	134.4
	Theileria parva	Q4N1X9_THEPA	3.90E-05	43.9
**Cysteine Proteinase Falcipain 2b (FP2B)**	*Theileria parva*	Q4N068_THEPA	1.20E-49	191.8
	Theileria parva	Q4N640_THEPA	1.20E-41	165.2
	Theileria parva	Q4N069_THEPA	9.20E-39	155.6
	Theileria parva	Q4N067_THEPA	7.80E-38	152.5
	Theileria parva	Q4N639_THEPA	1.40E-34	141.7
	Theileria parva	Q4N638_THEPA	2.40E-34	141
	Theileria parva	Q4N1X9_THEPA	3.00E-05	44.3
**Heme Detoxification Protein (HDP)**	*Theileria parva*	Q4N158_THEPA	8.20E-45	174.5
**Falcilysin (FLN)**	*Theileria parva*	Q4N5N0_THEPA	6.10E-124	439.9
	Theileria parva	Q4N5N3_THEPA	5.60E-85	310.5
**M1-family alanyl aminopeptidase (M1AAP)**	*Theileria parva*	Q4N8R7_THEPA	1.20E-155	545
**M17 leucyl aminopeptidase (LAP)**	*Theileria parva*	Q4N216_THEPA	8.50E-90	325.5
**Dipeptidyl Aminopeptidase 1 (DPAP1)**	*Theileria parva*	Q4N638_THEPA	1.60E-07	52.4

P. falciparum Gene Query	Target Species Proteome	Protein ID	*e*-value	Bit Score
	Theileria parva	Q4N639_THEPA	1.60E-07	52.4
	Theileria parva	CYSP_THEPA	2.70E-07	51.6
**Kelch13 (K13)**	*Toxoplasma gondii*	A0A7J6K2F7_TOXGO	2.10E-95	346.7
**Ubiquitin Carboxyl-terminal Hydrolase 1 (UBP1)**	*Toxoplasma gondii*	A0A7J6JVT9_TOXGO	7.90E-55	214.2
**Ap2 Complex Subunit µ (Ap2-µ)**	*Toxoplasma gondii*	A0A7J6K2M7_TOXGO	1.10E-39	161.4
	Toxoplasma gondii	Q8MTC0_TOXGO	2.00E-17	87.4
	Toxoplasma gondii	Q1JSZ4_TOXGO	1.70E-16	84.3
**KIC7 (KIC7)**	*Toxoplasma gondii*	A0A7J6K2K4_TOXGO	2.40E-12	70.5
**EPS15-like Protein (EPS15)**	*Toxoplasma gondii*	A0A7J6K029_TOXGO	2.10E-21	101.3
**Cysteine Proteinase Falcipain 2a (FP2A)**	*Toxoplasma gondii*	Q6DMN0_TOXGO	9.60E-52	201.1
	Toxoplasma gondii	A0A7J6JWF7_TOXGO	7.90E-14	75.1
**Cysteine Proteinase Falcipain 2b (FP2B)**	*Toxoplasma gondii*	Q6DMN0_TOXGO	9.50E-52	201.1
	Toxoplasma gondii	A0A7J6JWF7_TOXGO	7.90E-14	75.1
**Plasmepsin II (PMII)**	*Toxoplasma gondii*	A0A7J6K902_TOXGO	1.30E-42	170.6
**Plasmepsin IV (PM4)**	*Toxoplasma gondii*	A0A7J6K902_TOXGO	2.10E-37	153.3
	Toxoplasma gondii	Q6PS96_TOXGO	1.90E-30	130.2
	Toxoplasma gondii	ASP3_TOXGO	1.60E-24	110.5
	Toxoplasma gondii	A0A7J6KGU9_TOXGO	6.60E-15	78.6
**Falcilysin (FLN)**	*Toxoplasma gondii*	A0A7J6K1V2_TOXGO	4.70E-116	416
**M1-family alanyl aminopeptidase (M1AAP)**	*Toxoplasma gondii*	A0A7J6K0L4_TOXGO	1.90E-169	593.2
		A0A7J6K120_TOXGO	1.70E-133	473.8
		A0A7J6KEL4_TOXGO	7.50E-129	458.4

P. falciparum Gene Query	Target Species Proteome	Protein ID	*e*-value	Bit Score
**M17 leucyl aminopeptidase (LAP)**	*Toxoplasma gondii*	A0A7J6KBK1_TOXGO	5.50E-113	404.8
**Aminopeptidase P (APP)**	*Toxoplasma gondii*	A0A7J6K387_TOXGO	5.60E-86	315.5
**Dipeptidyl Aminopeptidase 1 (DPAP1)**	*Toxoplasma gondii*	A0A7J6KAI0_TOXGO	1.10E-93	340.9
		A0A7J6K9D7_TOXGO	1.50E-74	277.3

S3 Table

Gene	Ortholog Code	PlasmoDB Gene ID	Species	Host Group
**Aminopeptidase P (APP)**	OG6_100896	Htart_000005900	*Haemoproteus tartakovskyi*	Birds
**Aminopeptidase P (APP)**	OG6_100896	PGAL8A_00213600	*Plasmodium gallinaceum*	Birds
**Aminopeptidase P (APP)**	OG6_100896	PRELSG_1226600	*Plasmodium relictum*	Birds
**Aminopeptidase P (APP)**	OG6_100896	PYYM_1318900	*Plasmodium yoelii*	Rodents
**Aminopeptidase P (APP)**	OG6_100896	PBANKA_1318100	*Plasmodium berghei*	Rodents
**Aminopeptidase P (APP)**	OG6_100896	PCHAS_1321400	*Plasmodium chabaudi*	Rodents
**Aminopeptidase P (APP)**	OG6_100896	PF3D7_1454400	*Plasmodium falciparum*	Primates
**Aminopeptidase P (APP)**	OG6_100896	PRG01_1454400	*Plasmodium reichenowi*	Primates
**Aminopeptidase P (APP)**	OG6_100896	PGABG01_1453200	*Plasmodium gaboni*	Primates
**Ap2 Complex Subunit μ (Ap2-μ)**	OG6_138668	Htart_000099700	*Haemoproteus tartakovskyi*	Birds
**Ap2 Complex Subunit μ (Ap2-μ)**	OG6_138668	PGAL8A_00532900	*Plasmodium gallinaceum*	Birds
**Ap2 Complex Subunit μ (Ap2-μ)**	OG6_138668	PRELSG_1434400	*Plasmodium relictum*	Birds
**Ap2 Complex Subunit μ (Ap2-μ)**	OG6_138668	PY17X_1436300	*Plasmodium yoelii*	Rodents
**Ap2 Complex Subunit μ (Ap2-μ)**	OG6_138668	PBANKA_1433900	*Plasmodium berghei*	Rodents
**Ap2 Complex Subunit μ (Ap2-μ)**	OG6_138668	PCHAS_1435900	*Plasmodium chabaudi*	Rodents
**Ap2 Complex Subunit μ (Ap2-μ)**	OG6_138668	PF3D7_1218300	*Plasmodium falciparum*	Primates
**Ap2 Complex Subunit μ (Ap2-μ)**	OG6_138668	PRCDC_1217600	*Plasmodium reichenowi*	Primates
**Ap2 Complex Subunit μ (Ap2-μ)**	OG6_138668	PGABG01_1217400	*Plasmodium gaboni*	Primates
**Dipeptidyl Aminopeptidase 1 (DPAP1)**	OG6_103622	Htart_000103000	*Haemoproteus tartakovskyi*	Birds
**Dipeptidyl Aminopeptidase 1 (DPAP1)**	OG6_103622	PGAL8A_00343300	*Plasmodium gallinaceum*	Birds
**Dipeptidyl Aminopeptidase 1 (DPAP1)**	OG6_103622	PRELSG_0913500	*Plasmodium relictum*	Birds
**Dipeptidyl Aminopeptidase 1 (DPAP1)**	OG6_103622	PYYM_0932700	*Plasmodium yoelii*	Rodents
**Dipeptidyl Aminopeptidase 1 (DPAP1)**	OG6_103622	PBANKA_0931300	*Plasmodium berghei*	Rodents
**Dipeptidyl Aminopeptidase 1 (DPAP1)**	OG6_103622	PCHAS_0913000	*Plasmodium chabaudi*	Rodents
**Dipeptidyl Aminopeptidase 1 (DPAP1)**	OG6_103622	PF3D7_1116700	*Plasmodium falciparum*	Primates
**Dipeptidyl Aminopeptidase 1 (DPAP1)**	OG6_103622	PRCDC_1115100	*Plasmodium reichenowi*	Primates

Gene	Ortholog Code	PlasmoDB Gene ID	Species	Host Group
**EPS15-like Protein (EPS15)**	OG6_101578	Htart_000067200	*Haemoproteus tartakovskyi*	Birds
**EPS15-like Protein (EPS15)**	OG6_101578	PGAL8A_00484300	*Plasmodium gallinaceum*	Birds
**EPS15-like Protein (EPS15)**	OG6_101578	PRELSG_0608700	*Plasmodium relictum*	Birds
**EPS15-like Protein (EPS15)**	OG6_101578	PY17X_0510300	*Plasmodium yoelii*	Rodents
**EPS15-like Protein (EPS15)**	OG6_101578	PBANKA_0509200	*Plasmodium berghei*	Rodents
**EPS15-like Protein (EPS15)**	OG6_101578	PCHAS_0509300	*Plasmodium chabaudi*	Rodents
**EPS15-like Protein (EPS15)**	OG6_101578	PF3D7_1025000	*Plasmodium falciparum*	Primates
**EPS15-like Protein (EPS15)**	OG6_101578	PRCDC_1024400_	*Plasmodium reichenowi*	Primates
**EPS15-like Protein (EPS15)**	OG6_101578	PGABG01_1023000	*Plasmodium gaboni*	Primates
**Falcilysin (FLN)**	OG6_101809	Htart_000086200	*Haemoproteus tartakovskyi*	Birds
**Falcilysin (FLN)**	OG6_101809	PGAL8A_00167200	*Plasmodium gallinaceum*	Birds
**Falcilysin (FLN)**	OG6_101809	PRELSG_1109900	*Plasmodium relictum*	Birds
**Falcilysin (FLN)**	OG6_101809	PY17X_1138400	*Plasmodium yoelii*	Rodents
**Falcilysin (FLN)**	OG6_101809	PBANKA_1137000	*Plasmodium berghei*	Rodents
**Falcilysin (FLN)**	OG6_101809	PCHAS_1136500	*Plasmodium chabaudi*	Rodents
**Falcilysin (FLN)**	OG6_101809	PF3D7_1360800	*Plasmodium falciparum*	Primates
**Falcilysin (FLN)**	OG6_101809	PRG01_1363100	*Plasmodium reichenowi*	Primates
**Falcilysin (FLN)**	OG6_101809	PGABG01_1358500	*Plasmodium gaboni*	Primates
**Heme Detoxification Protein (HDP)**	OG6_139254	Htart_000070100	*Haemoproteus tartakovskyi*	Birds
**Heme Detoxification Protein (HDP)**	OG6_139254	PGAL8A_00206000	*Plasmodium gallinaceum*	Birds
**Heme Detoxification Protein (HDP)**	OG6_139254	PRELSG_1234200	*Plasmodium relictum*	Birds
**Heme Detoxification Protein (HDP)**	OG6_139254	PY17X_1314400	*Plasmodium yoelii*	Rodents
**Heme Detoxification Protein (HDP)**	OG6_139254	PBANKA_1310600	*Plasmodium berghei*	Rodents
**Heme Detoxification Protein (HDP)**	OG6_139254	PCHAS_1313900	*Plasmodium chabaudi*	Rodents
**Heme Detoxification Protein (HDP)**	OG6_139254	PF3D7_1446800	*Plasmodium falciparum*	Primates
**Heme Detoxification Protein (HDP)**	OG6_139254	PRG01_1446800	*Plasmodium reichenowi*	Primates
**Heme Detoxification Protein (HDP)**	OG6_139254	PGABG01_1445600	*Plasmodium gaboni*	Primates
**Kelch13 (K13)**	OG6_119343	Htart_000117800	*Haemoproteus tartakovskyi*	Birds
**Kelch13 (K13)**	OG6_119343	PGAL8A_00254800	*Plasmodium gallinaceum*	Birds

Gene	Ortholog Code	PlasmoDB Gene ID	Species	Host Group
**Kelch13 (K13)**	OG6_119343	PRELSG_1254200	*Plasmodium relictum*	Birds
**Kelch13 (K13)**	OG6_119343	PY17X_1362400	*Plasmodium yoelii*	Rodents
**Kelch13 (K13)**	OG6_119343	PBANKA_1356700	*Plasmodium berghei*	Rodents
**Kelch13 (K13)**	OG6_119343	PCHAS_1361300	*Plasmodium chabaudi*	Rodents
**Kelch13 (K13)**	OG6_119343	PF3D7_1343700	*Plasmodium falciparum*	Primates
**Kelch13 (K13)**	OG6_119343	PRG01_1346200	*Plasmodium reichenowi*	Primates
**Kelch13 (K13)**	OG6_119343	PGABG01_1341700	*Plasmodium gaboni*	Primates
**M1-family alanyl aminopeptidase (M1AAP)**	OG6_106799	Htart_000160100	*Haemoproteus tartakovskyi*	Birds
**M1-family alanyl aminopeptidase (M1AAP)**	OG6_106799	PGAL8A_00508000	*Plasmodium gallinaceum*	Birds
**M1-family alanyl aminopeptidase (M1AAP)**	OG6_106799	PRELSG_1409700	*Plasmodium relictum*	Birds
**M1-family alanyl aminopeptidase (M1AAP)**	OG6_106799	PY17X_1412100	*Plasmodium yoelii*	Rodents
**M1-family alanyl aminopeptidase (M1AAP)**	OG6_106799	PBANKA_1410300	*Plasmodium berghei*	Rodents
**M1-family alanyl aminopeptidase (M1AAP)**	OG6_106799	PCHAS_1412200	*Plasmodium chabaudi*	Rodents
**M1-family alanyl aminopeptidase (M1AAP)**	OG6_106799	PF3D7_1311800	*Plasmodium falciparum*	Primates
**M1-family alanyl aminopeptidase (M1AAP)**	OG6_106799	PRG01_1313900	*Plasmodium reichenowi*	Primates
**M1-family alanyl aminopeptidase (M1AAP)**	OG6_106799	PGABG01_1309900	*Plasmodium gaboni*	Primates
**M17 leucyl aminopeptidase (LAP)**	OG6_100682	Htart_000020000	*Haemoproteus tartakovskyi*	Birds
**M17 leucyl aminopeptidase (LAP)**	OG6_100682	PGAL8A_00205400	*Plasmodium gallinaceum*	Birds
**M17 leucyl aminopeptidase (LAP)**	OG6_100682	PRELSG_1234800	*Plasmodium relictum*	Birds
**M17 leucyl aminopeptidase (LAP)**	OG6_100682	PY17X_1313800	*Plasmodium yoelii*	Rodents
**M17 leucyl aminopeptidase (LAP)**	OG6_100682	PBANKA_1309900	*Plasmodium berghei*	Rodents
**M17 leucyl aminopeptidase (LAP)**	OG6_100682	PCHAS_1313100	*Plasmodium chabaudi*	Rodents
**M17 leucyl aminopeptidase (LAP)**	OG6_100682	PF3D7_1446200	*Plasmodium falciparum*	Primates
**M17 leucyl aminopeptidase (LAP)**	OG6_100682	PRG01_1446200	*Plasmodium reichenowi*	Primates
**M17 leucyl aminopeptidase (LAP)**	OG6_100682	PGABG01_1445000	*Plasmodium gaboni*	Primates
**Plasmepsin IV (PM4)**	OG6_100536	Htart_000153600	*Haemoproteus tartakovskyi*	Birds
**Plasmepsin IV (PM4)**	OG6_100536	PGAL8A_00279700	*Plasmodium gallinaceum*	Birds
**Plasmepsin IV (PM4)**	OG6_100536	PRELSG_1343000	*Plasmodium relictum*	Birds
**Plasmepsin IV (PM4)**	OG6_100536	PY17X_1036800	*Plasmodium yoelii*	Rodents

Gene	Ortholog Code	PlasmoDB Gene ID	Species	Host Group
**Plasmepsin IV (PM4)**	OG6_100536	PBANKA_1034400	*Plasmodium berghei*	Rodents
**Plasmepsin IV (PM4)**	OG6_100536	PCHAS_1035200	*Plasmodium chabaudi*	Rodents
**Plasmepsin IV (PM4)**	OG6_100536	PF3D7_1407800	*Plasmodium falciparum*	Primates
**Plasmepsin IV (PM4)**	OG6_100536	PRG01_1407500	*Plasmodium reichenowi*	Primates
**Plasmepsin IV (PM4)**	OG6_100536	PGABG01_1406200	*Plasmodium gaboni*	Primates

S4 Table

Primer Name	Sequence (5' - 3')	Target	Citation
**Mal Uni F**	GTT CGC CGG GGA TAA CAG GTT ATA G	Amplification of mitochondrial ribosomal RNA sequences for Haemosporidian parasite detection	Unpublished-designed by S. L. Perkins
**Mal Uni R**	ATA CAG TCC CAG CGA CAG CGG TTA T	Amplification of mitochondrial ribosomal RNA sequences for Haemosporidian parasite detection	Unpublished-designed by S. L. Perkins
**HAEMF**	ATG GTG CTT TCG ATA TAT GCA TG	Amplification of mitochondrial gene cytochrome b fragment; designed to be universal across avian *Leucocytozoon*, *Haemoproteus*, and *Plasmodium* species	Hellgren *et al.* 2004 (90)
**HAEMR2**	GCA TTA TCT GGA TGT GAT AAT GGT	Amplification of mitochondrial gene cytochrome b fragment; designed to be universal across avian *Leucocytozoon*, *Haemoproteus*, and *Plasmodium* species	Hellgren *et al.* 2004 (90)
**DW2**	TAA TGC CTA GAC GTA TTC CTG ATT ATC CAG	Amplification of *Plasmodium* species mitochondrial gene cytochrome b fragment	Creasey *et al.* 1993 (91)
**DW4**	TGT TTG CTT GGG AGC TGT AAT CAT AAT GTG	Amplification of *Plasmodium* species mitochondrial gene cytochrome b fragment	Creasey *et al.* 1993 (91)
**DW6**	GGG AGC TGT AAT CAT AAT GTG	Amplification of *Plasmodium* species mitochondrial gene cytochrome b fragment	Creasey *et al.* 1993 (91)
**DW8**	GCA CAA ATC CTT TAG GGT ATG ATA C	Amplification of *Plasmodium* species mitochondrial gene cytochrome b fragment	Perkins and Schall 2002 (92)
**3932F**	GGG TTA TGT ATT ACC TTG GGG TC	Amplification of *Plasmodium* species mitochondrial gene cytochrome b fragment	Perkins and Austin 2009 (93)
**3932R**	GAC CCC AAG GTA ATA CAT AAC CC	Amplification of *Plasmodium* species mitochondrial gene cytochrome b fragment	Perkins and Austin 2009 (93)
**COIF**	CTA TTT ATG GTT TTC ATT TTT ATT TGG TA	Amplification of *Plasmodium* species mitochondrial gene cytochrome oxidase I fragment	Perkins and Austin 2009 (93)
**COIR**	GTA TTT TCT CGT AAT GTT TTA CCA AAG AA	Amplification of *Plasmodium* species mitochondrial gene cytochrome oxidase I fragment	Perkins and Austin 2009 (93)
**COIinF**	ATG ATA TTT ACA RTT CAY GGW ATT ATT ATG	Amplification of *Plasmodium* species mitochondrial gene cytochrome oxidase I fragment	Perkins and Austin 2009 (93)
**COIinR**	GTA TTT TCT CGT AAT GTT TTA CCA AAG AA	Amplification of *Plasmodium* species mitochondrial gene cytochrome oxidase I fragment	Perkins and Austin 2009 (93)

Primer Name	Sequence (5' - 3')	Target	Citation
**COImidF**	TTA TTC TGG TTT TTT GGT CAT CCA G	Amplification of *Plasmodium* species mitochondrial gene cytochrome oxidase I fragment	Perkins and Austin 2009 (93)
**COImidR**	CTG GAT GAC CAA AAA ACC AGA ATA A	Amplification of *Plasmodium* species mitochondrial gene cytochrome oxidase I fragment	Perkins and Austin 2009 (93)
**CytbAE298-EF**	TGTAATGCCTAGACGTATTCC	Amplification of mitochondrial gene cytochrome b for avian Haemosporidian parasite detection	Pacheco *et al.* 2018 (94)
**CytbAE299-ER**	GTCAAWCAAACATGAATATAGAC	Amplification of mitochondrial gene cytochrome b for avian Haemosporidian parasite detection	Pacheco *et al.* 2018 (94)
**CytbAE064-IF**	TCTATTAATTTAGYWAAAGCAC	Amplification of mitochondrial gene cytochrome b for avian Haemosporidian parasite detection	Pacheco *et al.* 2018 (94)
**CytbAE066-IR**	GCTTGGGAGCTGTAATCATAAT	Amplification of mitochondrial gene cytochrome b for avian Haemosporidian parasite detection	Pacheco *et al.* 2018 (94)
**CytbAE974-EF**	TGTAATGCCTAGAMGWATWCC	Amplification of mitochondrial gene cytochrome b for avian Haemosporidian parasite detection	Pacheco *et al.* 2018 (94)
**CytbAE299-ER**	GTCAAWCAAACATGAATATAGAC	Amplification of mitochondrial gene cytochrome b for avian Haemosporidian parasite detection	Pacheco *et al.* 2018 (94)
**Cox3AE959-F**	CCATACAATYTCNACRAAATGCC	Amplification of mitochondrial gene cytochrome b for avian Haemosporidian parasite detection	Pacheco *et al.* 2018 (94)
**Cox3AE961-R**	CTGTTATCCCCGGCGAACC	Amplification of mitochondrial gene cytochrome b for avian Haemosporidian parasite detection	Pacheco *et al.* 2018 (94)
**PlasMit1989F**	CCT GAC ATG GAT GGA TAA TAC TCG	Designed for whole mitochondrial genome amplification for *Plasmodium* species	Perkins 2008 (95)
**PlasMit1989R**	GCG TTA AAA GCG TTC GTT CTT	Designed for whole mitochondrial genome amplification for *Plasmodium* species	Perkins 2008 (95)
**PlasMit1651F**	TCT CAT CGC AGC CTT GCA AT	Designed for whole mitochondrial genome amplification for *Plasmodium* species	Perkins 2008 (95)
**PlasMit1651R**	ACG ACA TGG AGG TGC CAA TAG TAT ATA A	Designed for whole mitochondrial genome amplification for *Plasmodium* species	Perkins 2008 (95)
**PlasMit349F**	TCG CTT CTA ACG GTG AAC TCT CAT T	Designed for whole mitochondrial genome amplification for *Plasmodium* species	Perkins 2008 (95)
**PlasMit349R**	ATG CGT GAG CTG GGT TAA GAA C	Designed for whole mitochondrial genome amplification for *Plasmodium* species	Perkins 2008 (95)

Primer Name	Sequence (5' - 3')	Target	Citation
**Pleuc349rF**	GAC ATC GAT ATA CGG ATT TCT CC	Forward primer to fill in gaps in the mitochondrial genome sequence, designed from *P. leucocytica* 349R sequence (primers above)	This Study
**Pleuc349rR**	GAA TAG AAA CAG ATG CCA GGC	Reverse primer to fill in gaps in the mitochondrial genome sequence, designed from *P. leucocytica* 349R sequence (primers above)	This Study
**Pleuc1989rF**	GTATTAATGACGCTGTGTTAGG	Forward primer to fill in gaps in the mitochondrial genome sequence, designed from *P. leucocytica* 1989R sequence (primers above)	This Study
**Pleuc1989rR**	GAGTTGAGATGGAAACAGCC	Reverse primer to fill in gaps in the mitochondrial genome sequence, designed from *P. leucocytica* 1989R sequence (primers above)	This Study
**Pleuc1989fF**	GCAATGAGCTGTATAGCCG	Forward primer to fill in gaps in the mitochondrial genome sequence, designed from *P. leucocytica* 1989F sequence (primers above)	This Study
**Pleuc1989fR**	GAGGCTTGGATATGAGTGAG	Reverse primer to fill in gaps in the mitochondrial genome sequence, designed from *P. leucocytica* 1989F sequence (primers above)	This Study
**Pleuc3932fF**	GATTACAGCCTCCCAAGC	Forward primer to fill in gaps in the mitochondrial genome sequence, designed from *P. leucocytica* 3932F sequence (primers above)	This Study
**Pleuc3932fR**	GAACAATATGTAAAGGAGTAGC	Forward primer to fill in gaps in the mitochondrial genome sequence, designed from *P. leucocytica* 3932F sequence (primers above)	This Study
**Pleuc1989Rfwd**	GCATCTTGTATGACTGCATGTC	Primer to close gap in *P. leucocytica* mitochondrial genome, designed from 1989R sequence (primers above)	This Study
**Pflor349Rfwd**	CGTTATTAAGCGTCAGGAAGTC	Primer to close gap in *P. floridense* mitochondrial genome, designed from 349R sequence (primers above)	This Study
**Pflor349Frev**	GTTAGAAGCAAACACTAGCGG	Primer to close gap in *P. floridense* mitochondrial genome, designed from 349F sequence (primers above)	This Study

Primer Name	Sequence (5’ - 3’)	Target	Citation
**Pflor3932Ffwd**	GACATGATAGGGAGTTGGC	Primer to close gap in *P. floridense* mitochondrial genome, designed from 3932F sequence (primers above)	This Study
**Pazur349Rrev**	GGGGCAGATGTCAGTAACTTG	Primer to close gap in *P. azurophilum* mitochondrial genome, designed from 349R sequence (primers above)	This Study
**Pazur3932Ffwd**	GCTGTTGATGGATGCTTCG	Primer to close gap in *P. azurophilum* mitochondrial genome, designed from 349R sequence (primers above)	This Study
